# Characterisation of innate lymphoid cell populations at different sites in mice with defective T cell immunity

**DOI:** 10.12688/wellcomeopenres.13199.3

**Published:** 2018-03-14

**Authors:** Emma E. Dutton, Ana Camelo, Matthew Sleeman, Ronald Herbst, Gianluca Carlesso, Gabrielle T. Belz, David R. Withers

**Affiliations:** 1 Institute of Immunology and Immunotherapy, College of Medical and Dental Sciences, University of Birmingham, Birmingham, UK; 2 MedImmune, Aaron Klug building, Granta Park, Cambridge, UK; 3 Immunology & Inflammation Group, Regeneron, Tarrytown, NY, USA; 4 Department of Cancer Biology, MedImmune, Gaithersburg, MD, USA; 5 Department of Medical Biology, University of Melbourne, Parkville, Victoria, Australia; 6 The Walter and Eliza Hall Institute of Medical Research, Parkville, Victoria, Australia

**Keywords:** Innate Lymphoid Cells, T cells, CD80, CD86, ICOS, ICOSL

## Abstract

**Background**: Innate lymphoid cells (ILCs) have now been identified within most tissues of the body and current evidence indicates that this family of cells play a fundamental role in maintaining tissue homeostasis. However, few studies have compared the ILC populations between several tissues.

**Methods**: We sought to generate a comprehensive characterisation of the ILC populations in different tissues of C57BL/6 WT and genetically modified mice targeting costimulatory pathways, using transcription factor expression to define specific groups.

**Results**: Consistent with studies individually describing the ILC composition in different tissues, our analysis revealed different ILC groups dominate the ILC population in different tissues. Additionally, we observed a population of  IL-7Rα ^+^Id2 ^+^ cells lacking expression of lineage markers but also lacking expression of GATA-3, RORgt or T-bet. This population was most evident in ear skin where it outnumbered the defined ILC groups, however, further experiments demonstrated that detection of these cells was influenced by how the tissue was digested, raising concerns as to its real nature. Since both ILC2 and ILC3 express ICOS, we then investigated the requirement for ICOS:ICOSL interactions in the homeostasis of ILC populations at these sites. Surprisingly, no significant differences were detected in the number of ILC1, ILC2 or ILC3 between WT and ICOSL ^-/-^ mice in any tissue, indicating that this pathway is not required for ILC homeostasis at these sites. These data were compared with CD80 ^-/-^CD86 ^-/-^ mice given evidence of CD28 expression by some ILC and ILC crosstalk with activated T cells. Notably, the absence of CD28 ligands resulted in a significant increase in ILC2 and ILC3 numbers in the intestine.

**Conclusions**: Together, these data provide new insight into ILC composition in different tissues in both WT and genetically modified mice where key costimulatory pathways are genetically deleted, providing a useful resource for further research into ILC biology.

## Introduction

In recent years, innate lymphoid cells (ILCs) have received significant attention and current evidence indicates they play a key role in the maintenance of tissue homeostasis and the initiation of inflammatory responses ^1^. Whilst initial studies of ILCs focused on the intestine, it is now evident that these cells are located in most, if not all tissues in the body, including mucosal barrier sites, primary and secondary lymphoid tissue, skin and organs such as the liver, heart and bladder ^2– 4^. Formal nomenclature of the ILC groups brought together key phenotypic and functional data and provided a clear framework in which to further investigate this family of immune cells ^5^. Three distinct groups of ILCs, characterised on the basis of transcription factor expression and associated cytokine production have been described and whilst plasticity may blur our understanding of how different populations developed within a tissue ^6^, transcription factor expression has provided a robust means of unequivocally identifying ILC populations, superior to reliance on surface markers that show tissue-specific variations in expression.

Although ILCs appear ubiquitous in the body, within given tissues it is evident that distinct ILC groups dominate. For example, whilst all ILCs are present in the intestinal tract, the majority of these cells belong to the RORγt-expressing group 3 population (ILC3) ^6, 7^. In contrast, the vast majority of ILCs in lung or adipose tissue express high levels of GATA-3 characteristic of the group 2 ILCs (ILC2) ^8– 11^. Furthermore, developmentally distinct subsets of ILC within a specific group are evident, particularly amongst ILC3s and these also exhibit tissue tropism ^12^. Our previous analysis of ILC populations within secondary lymphoid tissues relied on transcription factor expression rather than surface markers and this approach clearly identified three distinct ILC groups that accounted for the total IL-7Rα ^+^Lineage ^-^ (Lin ^-^) population of cells ^13^. Thus our aim was to extend this approach to compare ILC populations within a range of lymphoid and non-lymphoid tissues, reasoning that this would provide a useful resource from which to better develop understanding of ILC populations. Several studies have also indicated that ILC:T cell cross talk shapes the outcome of CD4 T cell responses ^7, 14, 15^ and that both cell types require common γ chain cytokines for their homeostasis ^16– 18^. Furthermore, ILCs express a number of costimulatory molecules whose expression is shared with T cells, although the role these molecules play in ILC function requires further investigation ^3, 19, 20^. Thus, the secondary aim of this work was to characterise ILCs in mice in which specific costimulatory molecules were absent.

## Methods

### Mice

All mice used were bred and maintained in accordance with Home Office guidelines at the University of Birmingham, Biomedical Services Unit. Mice used were SPF status on a C57BL/6 background, including CD80 ^-/-^86 ^-/-^^21^, ICOS ^-/-^ (provided by Medimmune) ^22^, ICOSL ^-/-^^23^, Id2-eGFP ^24^ and WT. All mice were used at 6 weeks of age or older (up to 12 weeks of age). Within experiments, mice were age and sex matched, but not randomised. Mice were housed within IVC cages at up to 4 mice per cage of the same sex dependent upon the composition of the litter. Mice were checked daily by trained staff and every effort was made to ameliorate any suffering of the animals. Mice were checked daily by trained staff. Mice were culled by cervical dislocation.

### Cell isolation

***Secondary lymphoid tissue digestion protocol.*** Lymph nodes (LN) were cleaned and teased in Roswell Park Memorial Institute (RPMI) 1640 Medium (life technologies). For digestion, LNs were incubated at 37°C for 20 minutes in collagenase dispase (final concentration, 0.25 mg/ml) (Roche Life Sciences) and DNAse (0.025 mg/ml) (Roche Diagnostics). Digestion was stopped through the addition of Ethylenediaminetetraacetic acid (EDTA) (0.01 M) (Sigma-Aldrich) and the tissues were crushed through a 70 μm filter. Samples were centrifuged (5minutes, 1,500 rpm, 4°C) and supernatant removed. LNs were resuspended in appropriate amount of DPBS supplemented with 2% FBS and 0.5% EDTA.

***Lung digestion protocol.*** Prior to the removal of the lungs they were perfused with DPBS (Life Technologies). Briefly, the right atrium of the heart was pierced and the left ventricle injected with 10 ml of DPBS, inflating the lungs and flushing out the blood. The lung was cleaned and teased apart in RPMI-1640 supplemented with 1% Penicillin Streptomycin solution, 1% L-Glutamine and 10% Fetal Bovine Serum (culture medium). Liberase TM/DNase solution (for one lung; 500 μL Liberase TM (42.4 μg/ml) (Roche Life Sciences), 10 μL DNAse (10 mg/ml) and 3.5 ml of culture media) was added per sample for digestion. The tissue was incubated at 37°C and shaken for 45 minutes before being crushed through a 70 μm filter and washed with culture media. Samples were then centrifuged, supernatant removed, resuspended in Gey’s red blood cell lysis buffer (70% H 2O, 20% solution A, 5% solution B, 5% solution C) ( Table 1) and incubated on ice for 2 minutes, before being diluted in culture media and then filtered, washed and re-suspended in an appropriate amount of DPBS supplemented with 2% FBS and 0.5% EDTA.

**Table 1.** Geys Red Cell Lysis Buffer.

Solution	Additive	Quantity
A	NH _4_Cl	35g
	KCl	1.85g
	Na _2_HPO _4_.12H _2_O	1.5g
	KH _2_PO _4_	0.119g
	Glucose	5.0g
	Gelatin	25.0g
	1% Phenol red	1.5ml
B	MgCl _2_.6H _2_O	4.2g
	MgSO _4_.7H _2_O	1.4g
	CaCl _2_	3.4g
C	NaHCO _5_	22.5g

***Small intestine digestion protocol.*** The small intestine (SI) was dissected from below the stomach and above the caecum then placed in a petri dish containing Hank’s Balanced Salt Solution (HBSS) (Sigma-Aldrich) 2% FBS. The fat and Peyer’s patches were removed before the SI was cut longitudinally and the contents washed out. The tissue was cut into small pieces, placed in HBSS and shaken vigorously, before being filtered through nitex mesh. The SI underwent a series of incubations in various digestion media; it was placed in a specific digestion media, shaken vigorously for 20 seconds, incubated and shaken at 37°C for 20 min (HBSS/EDTA wash) or 15 min (collagenase digestion). This is followed by a washing process where the SI was filtered, resuspended in HBSS and vigorously shaken for a further 20 seconds before being filtered again. This process was carried out with the following digestion media: HBSS 2mM EDTA (20 minutes) twice and pre-warmed culture media containing 1 mg/mL collagenase VIII (Sigma-Aldrich) (15 minutes). The SI was filtered through 100 μm and 70 μm cell strainers before being centrifuged and re-suspended in an appropriate amount of DPBS supplemented with 2% FBS and 0.5% EDTA.

***Ear skin digestion protocol.*** Ears were cut into small sections and incubated in a shaker at 37°C for 30 minutes in 1 ml of Liberase TM/DNase digestion solution (3 ml per ear; 3 ml DMEM, 75μL Liberase TM (0.28 Wunsch units/mL) and 75 μL DNase (200 mg/mL). The tissue was filtered through a 70 μm filter and washed with DMEM, and the supernatant collected. The ear was then removed from the strainer and this process repeated twice more. After the third incubation, the ear was crushed through the filter and washed with DMEM. Samples were centrifuged and suspended in an appropriate amount of DPBS supplemented with 2% FBS and 0.5% EDTA.

### Flow cytometry

Samples were stained using the antibodies in Table 2. A minimum of ^1^/ _6_ of cell suspensions were stained. To identify in ILCs dead cells were excluded using a LIVE/DEAD cell viability assay in APC-Cy7. A lineage cocktail containing monoclonal Abs to B220, CD11c, CD11b, CD3 and CD5 was used except where stated and ILC were identified as IL-7Rα ^+^Lin ^-^. Surface staining was conducted with antibodies diluted in DPBS supplemented with 2% FBS and 0.5% EDTA at 4°C for 30minutes. Cells were fixed and intracellularly stained using the Foxp3 Staining Buffer Set (eBioscience, catalogue number 00-5523-00) according to manufactures instructions. Samples were run on the BD LSRFortessa ^TM^ X-20 (BD Biosciences) and data collected using BD FACSDiva Software (BD biosciences).

**Table 2.** Antibodies used for flow cytometry.

Antibody Specificity	Conjugate (dilution)	Clone	Concentration (mg/ml)	Manufacture	Host	Catalogue Number	Class
B220 (CD45R)	FITC (1:300)	RA3-6B2	0.5	eBioscience	Rat	11-0452-63	Monoclonal
CCR6 (CD196)	BV605 (1:100)	29-2L17	0.2	BioLegend	Armenian hamster	129819	Monoclonal
CD11b	FITC (1:300)	M1/70	0.5	eBioscience	Rat	11-0112-85	Monoclonal
CD11c	FITC (1:300)	N418	0.5	eBioscience	Armenian hamster	11-0114-85	Monoclonal
CD123	FITC (1:500)	5B11	0.2	eBioscience	Rat	11-1231-82	Monoclonal
CD19	FITC (1:100)	eBio1D3	0.2	eBioscience	Rat	11-0193-82	Monoclonal
CD25	BV650 (1:200)	PC61	0.05	BioLegend	Rat	102038	Monoclonal
CD3	FITC (1:100)	145-2C11	0.5	eBioscience	Armenian hamster	11-0031-85	Monoclonal
	AF700 (1:100)	eBio500A2	0.2	eBioscience	Syrian hamster	56-0033-82	Monoclonal
CD45.2	BV510 (1:100)	104	0.1	BioLegend	Mouse	109837	Monoclonal
	BV785 (1:200)	104	0.2	BioLegend	Mouse	109839	Monoclonal
CD49b	FITC (1:200)	DX5	0.2	eBioscience	Rat	11-5971-82	Monoclonal
CD5	FITC (1:100)	53-7.3	0.5	eBioscience	Rat	11-0051-85	Monoclonal
F4/80	FITC (1:200)	BM8	0.2	eBioscience	Rat	11-4801-82	Monoclonal
FcεRI	FITC (1:200)	MAR-1	0.2	eBioscience	Armenian hamster	11-5898-82	Monoclonal
GATA-3	PerCP-eFluor 710 (1:50)	TWAJ	-	eBioscience	Rat	46-9966-42	Monoclonal
Gr-1	FITC (1:2000)	RB6-8C5	0.2	eBioscience	Rat	11-5931-82	Monoclonal
ICOS	PE-Cyanine7 (1:200)	C398.4A	0.2	BioLegend	Armenian hamster	313519	Monoclonal
IL-7R⍺	BV421 (1:100)	A7R34	0.1	BioLegend	Rat	135024	Monoclonal
KLRG1	APC (1:200)	2F1	0.2	eBioscience	Syrian hamster	17-5893-82	Monoclonal
MHCII	BV510 (1:500)	M5/114.15.2	0.1	BioLegend	Rat	107635	Monoclonal
NKp46 (CD335)	PE-Cyanine7 (1:100)	29A1.4	0.2	eBioscience	Rat	25-3351-82	Monoclonal
RORγt	PE (1:50)	AFKJS-9	0.2	eBioscience	Rat	12-6988-82	Monoclonal
ST2 (IL-33Rα)	PE (1:50)	DIH9	0.2	BioLegend	Rat	145304	Monoclonal
T-bet	eFluor 660 (1:50)	eBio4B10	0.2	eBioscience	Mouse	50-5825-82	Monoclonal
TER-119	FITC (1:100)	TER-119	0.2	eBioscience	Rat	11-5921-82	Monoclonal

### Statistical analysis

Data collected were analysed using Flow Jo software vX.0.7 (Treestar) and GraphPad Prism 6. Pairs of samples were compared using a two-tailed Mann-Whitney T test; multiple samples were compared using Kruskal-Wallis one-way ANOVA with post hoc Dunn’s test: * p ≤ 0.05, ** p ≤0.01, *** p ≤ 0.005. The bar represents the median and where appropriate the median has been shown. Where a p-value is not indicated, no statistical difference was observed.

## Results

### Characterisation of ILC populations in different lymphoid and non-lymphoid tissues

**Figure 1.**
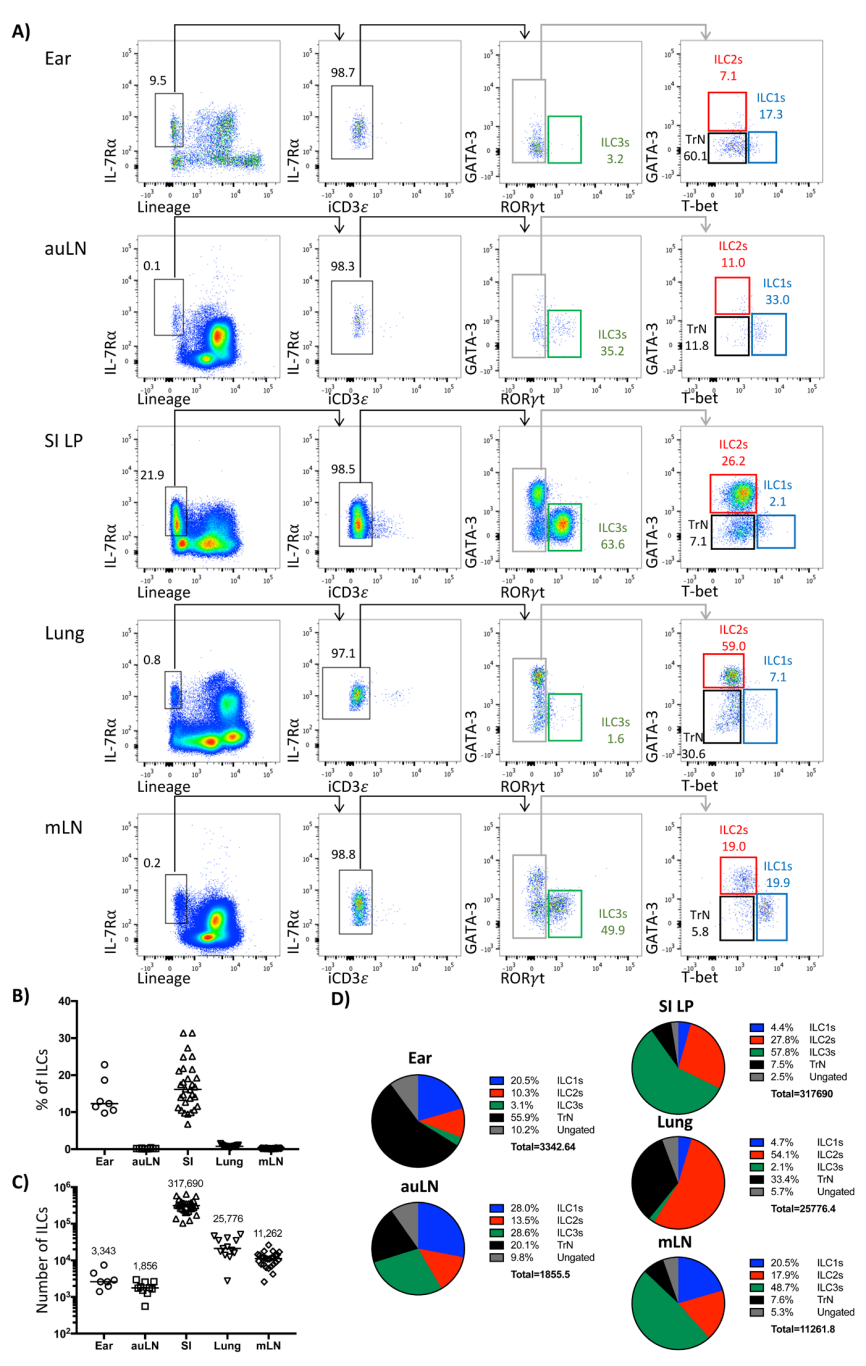
Identification of ILC groups in different tissues using transcription factor expression. To characterise ILC groups in lymphoid and non-lymphoid tissues, cells were isolated from the ear, auLN, SI LP, lung and mLN and the ILC groups identified on the basis of expression of the transcription factors GATA-3, RORγt and T-bet. **A**) Representative flow cytometry plots showing the gating approach used to identify the populations in the different tissues. ILCs were identified as live CD45 ^+^IL-7Rα ^+^Lin (CD3, CD5, B220, CD11b, CD11c) ^-^iCD3 ^-^ cells. Coloured gates used to show specific groups of cells with ILC1 (T-bet ^+^, blue), ILC2 (GATA-3 ^+^, red), ILC3 (RORγt ^+^, green) and ‘Triple Negative’ cells lacking expression of the GATA-3, RORγt and T-bet (black). **B**) ILCs as a percentage of the hematopoietic population in each tissue. **C**) The total numbers of ILCs isolated from each tissue. **D**) Pie charts showing the mean proportion of each ILC group using the group-specific colours used in part ‘ **A**)’. Cells falling outside of the transcription factor gates were termed ‘ungated’ and included to allow analysis of the entire ILC compartment. Each data point represents cells isolated from 2x ears, 2x auLNs, 1x SI LP, 1 x lung and 1 pool of mLNs isolated from one mouse. The data shown for ear (n=6), auLN (n=10), SI LP (n = 30), lung (n = 13) and mLN (n=26) are pooled from a minimum of 3 independent experiments for each tissue. Values on flow cytometry plots represent percentages, bars on scatter plots represents the median, which is also shown numerically for clarity.

Over the last few years, enormous progress has been made identifying ILC populations within different murine tissues. A range of surface markers were initially used to describe these cells, however, tissue specific differences in expression of these molecules is evident and has created some discrepancies in the populations described. We sought to assess all ILCs in a collection of non-lymphoid tissues such as the intestine, lung and peripheral skin and lymphoid (different lymph nodes) to better compare these populations at different sites. All ILC populations express IL-7Rα and we have previously found this a robust initial means of identifying ILC amongst cells lacking expression of markers associated with other lineages, with subsequent characterisation of ILC groups based largely on transcription factor expression ^13^. Thus, cells were prepared from SI lamina propria (SI LP), lung, ear skin, mesenteric LNs (mLN, a pool of 5 LNs per sample) and auricular LNs (auLN, a pool of 2 LNs per sample). The mLN were chosen to contrast with the SI LP and the auLN chosen to compare with the ear skin. ILCs were identified as IL-7Rα ^+^Lin ^- ^cells with cells expressing intracellular CD3ε (iCD3ε) also excluded to minimise potential T cell contamination of the ILC gate ( Figure 1A). Consistent with other studies, enumeration of ILCs within these different tissue identified that this family of cells was most numerous in the mucosal barrier sites (intestine and lung) as either a proportion of hematopoietic cells ( Figure 1B) or as total cell numbers per tissue ( Figure 1C). While a substantial ( ^~^10%) of the live hematopoietic cells within ear skin were located within the IL-7Rα ^+^Lin ^-^ gate, the number of CD45 ^+^ cells isolated from ear tissue was very low ( Figure 1B, C). A distinct ILC population was evident in the auLN and mLN, but numerically these were small populations and vastly out-numbered by naïve lymphocytes ^13^. Analysis of GATA-3, RORγt and T-bet expression amongst ILC clearly identified the three described ILC groups. In the SI LP and mLN this accounted for the vast majority of the IL-7Rα ^+^Lin ^- ^cells ( Figure 1A). Graphical representation of the mean proportion of each subset in each tissue from multiple mice illustrated the substantial differences in the proportion of ILC subtypes in the various tissues ( Figure 1D). In all the tissues analysed, but most evident within the ear skin, we detected a population of IL-7Rα ^+^Lin ^-^ cells lacking clear expression of GATA-3, RORγt and T-bet. We termed these cells ‘Triple Negative’ (TrN) cells in the context of this study. An obvious concern was that these cells were wrongly identified as ILC, due a failure to completely discriminate them on the basis of the lineage-defining antibodies used. Given the substantial proportion of these TrN cells identified in the ear skin, we repeated the analysis of ILC populations in ear skin using an extended cocktail of Abs against different immune cell lineages to potentially further restrict the cells termed IL-7Rα ^+^Lin ^-^ ( Supplementary Figure 1). Whilst this revised lineage discrimination modestly reduced the proportion of TrN cells, they remained a substantial population (almost 50%) of the IL-7Rα ^+^Lin ^-^ cells, arguing that these cells could not be accounted for solely through contamination with other immune cell populations. To provide evidence that these cells displayed further characteristics of ILCs, we assessed their expression of Id2 using Id2-eGFP reporter mice ^24^. The vast majority of IL-7Rα ^+^Lin ^- ^cells in the ear skin or draining auLN were eGFP ^+^ compared with WT controls ( Supplementary Figure 2), demonstrating that most of the TrN population expressed Id2, although levels of eGFP appeared lower than in splenic NK cells. However, a further concern was that the manner in which these cells were isolated was affecting the ILCs that could be identified. The protocol for the digestion of ear skin tissue uses the same enzymes used in the digestion of lung tissue, but at a higher concentration and for longer. To test whether the ear digestion protocol could affect the ILC populations identifiable by flow cytometry, we digested lung tissue with the normal lung digest protocol or the protocol used to digest the ear. Strikingly, the ear digestion protocol resulted in changes in the proportions of different ILC populations in the lung tissue, reducing the GATA-3 ^+^ population approximately 2-fold and doubling the proportion of TrN cells ( Supplementary Figure 3). We also assessed whether the ear digestion protocol was affecting any of the lineage markers detected by the cocktail of Abs used to identify ILCs or the individual expression of the transcription factors. Whilst some markers including CD4 and CD8 were clearly affected by the prolonged ear digestion, expression of the key lineage markers used to distinguish the IL-7Rα ^+^ population were not diminished, minimising the impact on ILC identification ( Supplementary Figure 4– Supplementary Figure 5). Notably, expression of GATA-3 and T-bet in cells isolated from the lung were reduced by the prolonged digestion used on ear tissue. Thus, while the TrN cells described here share several key features of ILCs, there are clear concerns that this population is either an artefact of the protocol used to isolate cells or at the very least likely exaggerated because of the digestion employed.

**Figure 2.**
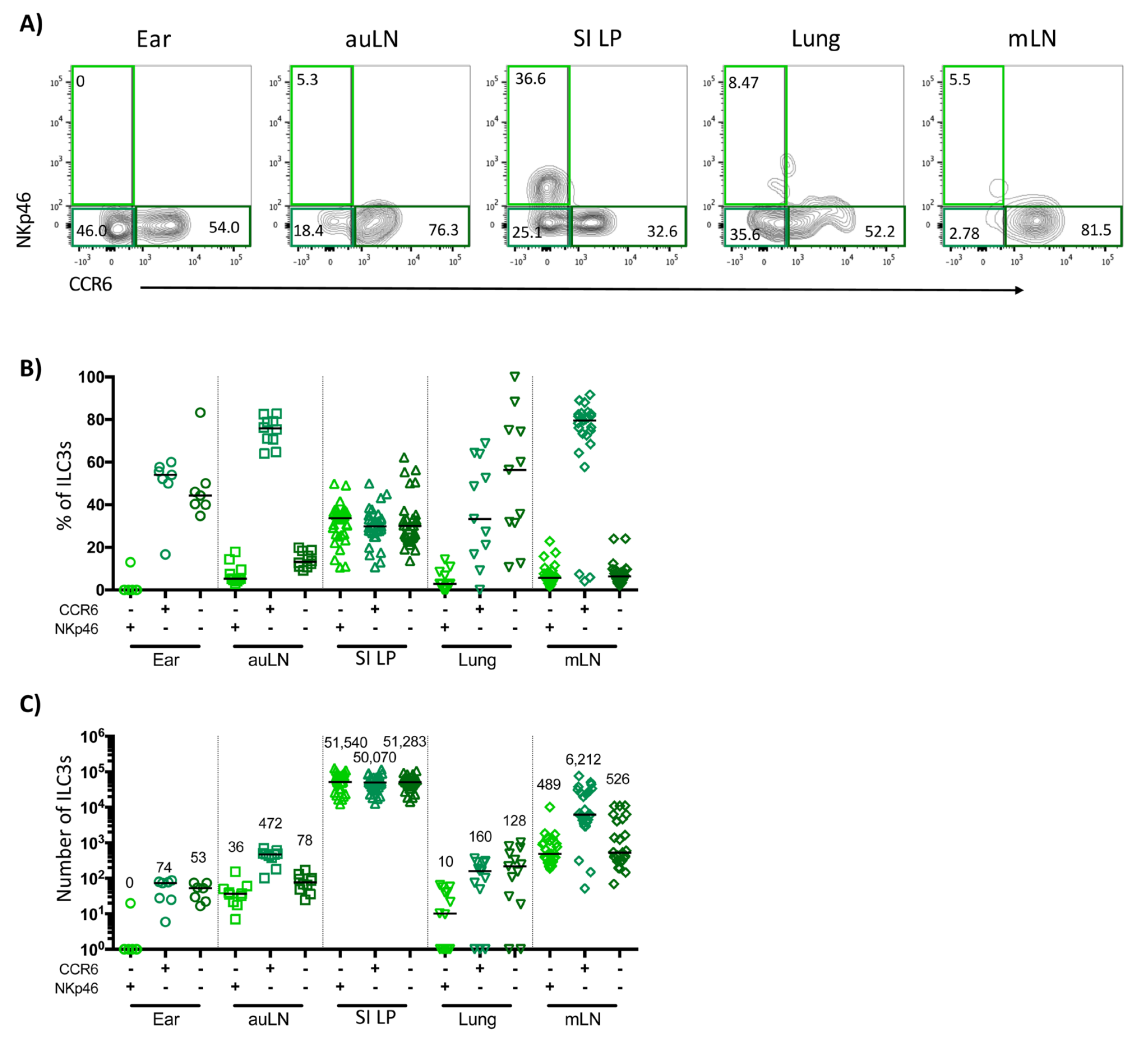
Identification of ILC3 subsets in different tissues using CCR6 and NKp46 expression. To identify the different subtypes of ILC3 in the tissues, RORγt expressing ILCs were assessed for expression of CCR6 and NKp46. **A**) Representative flow cytometry plots showing the gating approach used to identify the subsets in each tissue, having first gated on RORγt-expressing ILC3 as shown in Figure 1. **B**) The percentage of each ILC3 subset in the different tissues. **C**) The total numbers of each ILC3 subset isolated from each tissue. Each data point represents cells isolated from 2x ears, 2x auLNs, 1x SI LP, 1 x lung and 1 pool of mLNs isolated from one mouse. The data shown for ear (n=7), auLN (n=10), SI LP (n = 30), lung (n = 13) and mLN (n=26) are pooled from a minimum of 3 independent experiments for each tissue. Values on flow cytometry plots represent percentages, bars on scatter plots represents the median, which is also shown numerically for clarity.

It is now evident that ILC3s can be further split into developmentally distinct subsets based upon expression of CCR6 and NKp46, with CCR6 ^+^NKp46 ^-^ LTi-like ILC3, CCR6 ^-^NKp46 ^+^ ILC3 and a CCR6 ^-^NKp46 ^-^ population that contain cells able to differentiate into the CCR6 ^+^NKp46 ^-^ and CCR6 ^-^NKp46 ^+^ subsets ^6, 12^. Thus, CCR6 and NKp46 expression amongst the RORγt ^+^ ILC3 in the different tissues was analysed and consistent with other studies, all three populations were evident in the SI LP ( Figure 2A, B) and all populations were most numerous in this tissue compared with other sites ( Figure 2C). In the different LNs, the majority of the ILC3 were CCR6 ^+^Nkp46 ^-^, again consistent with previous work ^13^. Within the ear skin and lung, the number and proportion of ILC3 was very low, but this small population contained both CCR6 ^+^NKp46 ^-^ and CCR6 ^-^NKp46 ^-^ subsets. Thus, whilst most CCR6 ^-^NKp46 ^-^ ILC3 were present in the intestine, these were detected at low number in the other tissues. The NKp46 ^+^CCR6 ^-^ ILC3 appeared to be more restricted to the intestine than the other subsets of ILC3, in regard to the tissues assessed in this study.

**Figure 3.**
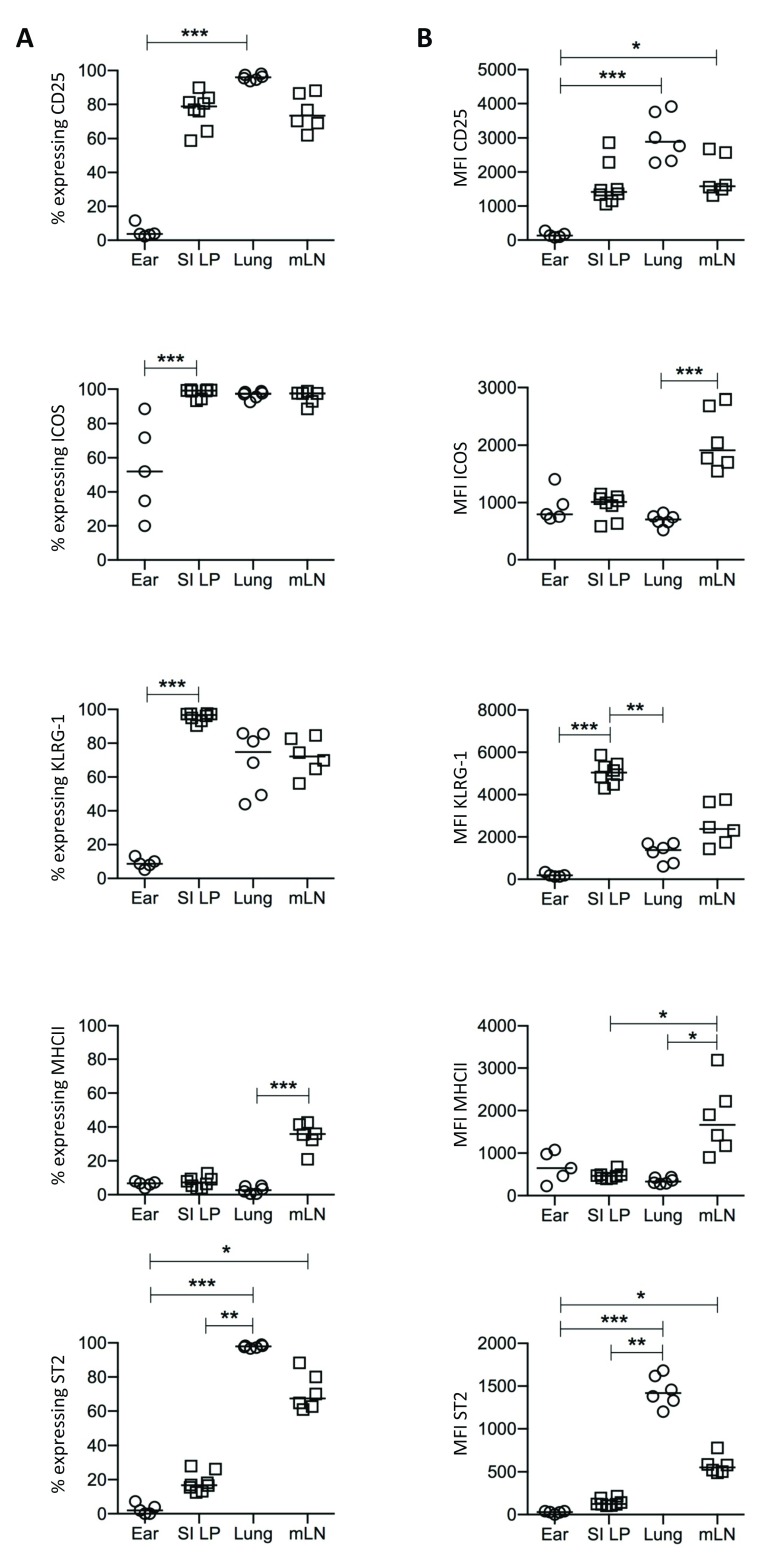
Characterisation of ILC2 surface phenotype in different tissues. To further characterise the ILC2 population identified in the different tissues, expression of CD25, ICOS, KLRG-1, MHCII and ST2 was assessed. **A**) The percentage of each GATA-3 ^+^ ILC expressing the stated surface marker isolated from ear skin, SI LP, lung or mLN. **B**) The median fluorescence intensity (MFI) of the stated surface marker total numbers of each ILC2 subset isolated from ear skin, SI LP, lung or mLN. Each data point represents cells isolated from 2x ears, 1x SI LP, 1 x lung and 1 pool of mLNs isolated from one mouse. The data shown for ear (n=5), SI LP (n = 8), lung (n = 6) and mLN (n=6) are pooled from 3 independent experiments for each tissue, bars on scatter plots represents the median. Statistical significance was tested using Kruskal-Wallis one-way ANOVA with post hoc Dunn’s test: *p≤0.05, **p≤0.01, ***p≤0.001.

To investigate the extent to which the tissue of origin affected their expression of reported surface markers, we further characterised the ILC2 in ear skin, lung, SI LP and mLN ( Figure 3, Supplementary Figure 6– Supplementary Figure 7). Whilst the most notable difference in phenotype was between ILC2 isolated from ear skin compared with other tissues, these data must be viewed with caution given the prolonged enzymatic digestion required to isolate cells from skin. It is evident that the digestion used to isolate cells from ear clearly reduces levels of KLRG-1 and CD25 versus the lung digestion protocol ( Supplementary Figure 6) so these expression data are flawed. ST2 expression did not appear to be reduced solely due to the digestion indicating that ILC2 in ear skin, as in the SI LP express less ST2. ILC2 have also been reported to express major histocompatibility complex class II (MHCII), facilitating interactions with CD4 T cells ^14^ and whilst ILC2 isolated from tissues had little detectable MHCII expression, amongst those isolated from the mLN, there appeared to be a clear MHCII ^+^ subset ( Figure 3A, B) ( Supplementary Figure 6– Supplementary Figure 7).

### Role of ICOS:ICOSL interactions in ILC homeostasis

**Figure 4.**
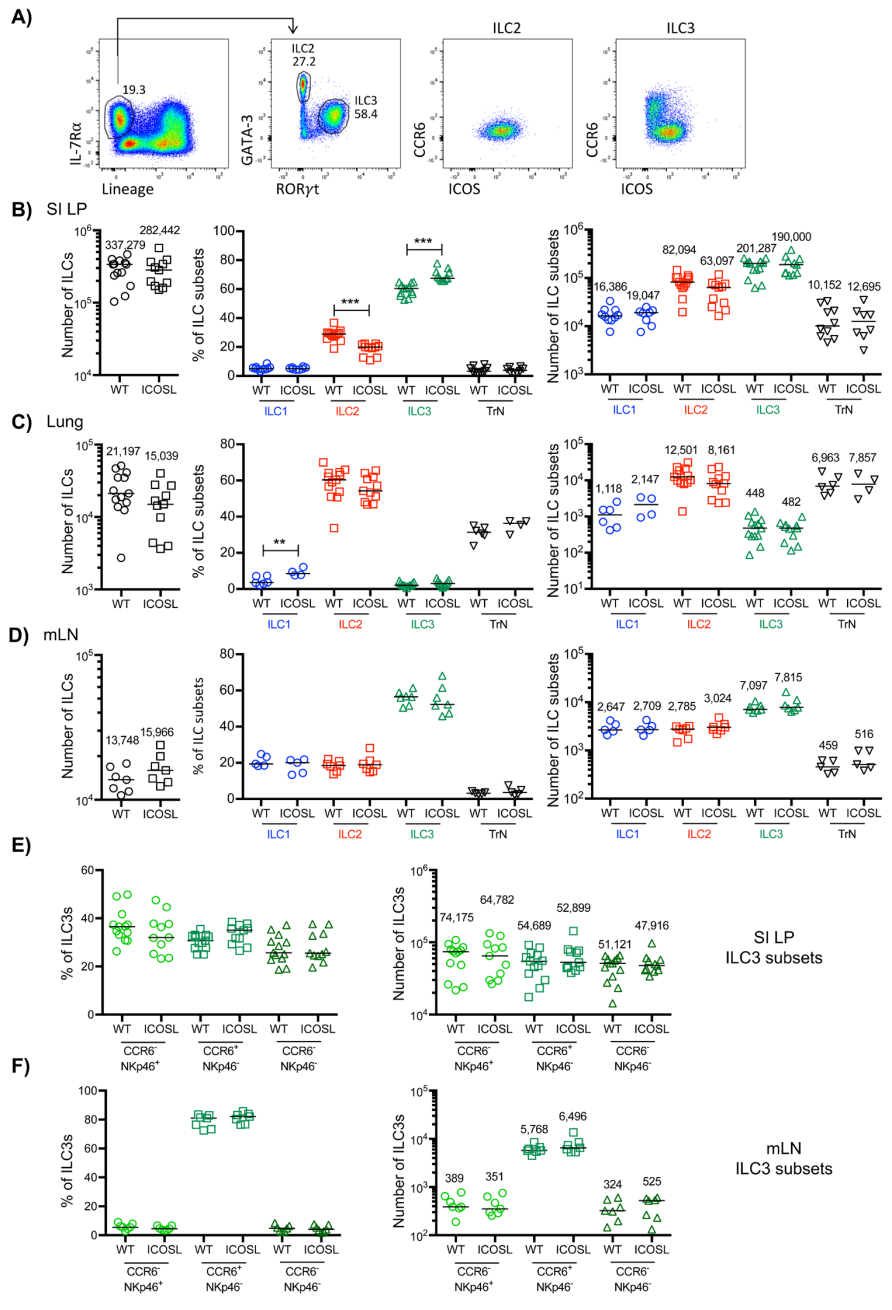
Normal numbers of ILC1, ILC2 and ILC3 in ICOSL ^-/-^ mice. To investigate the role of ICOS:ICOSL interactions in ILC homeostasis, cells were isolated from the SI LP, lung and mLNs of WT and ICOSL ^-/-^ mice and ILC groups enumerated. ILCs were identified as live CD45 ^+^IL-7Rα ^+^Lin (CD3, CD5, B220, CD11b, CD11c) ^-^iCD3 ^-^ cells, transcription factor expression was used to identify ILC1 (T-bet ^+^, blue), ILC2 (GATA-3 ^+^, red), ILC3 (RORγt ^+^, green) and ‘Triple Negative’ cells lacking expression of the GATA-3, RORγt and T-bet (black). **A**) Representative flow cytometry plots showing ICOS expression by GATA-3 ^+^ ILC2 and RORγt ^+^ ILC3 isolated from the SI LP of WT mice. **B**)– **D**) The total number of ILCs (left hand graph), the percentage of the ILCs within each group (centre graph) and the total number of each ILC group isolated from the SI LP ( **B**), lung ( **C**) and mLN ( **D**). **E**)– **F**) The percentage (left hand graph) and total number (right hand graph) of each ILC3 subset isolated from the SI LP ( **E**) and mLN ( **F**) of WT and ICOSL ^-/-^ mice. Each data point represents cells isolated from 1 x SI LP, 1 x lung and 1 pool of mLNs isolated from one mouse. The data shown for SI LP WT (n=10–13), SI LP ICOSL ^-/-^ (n=8–11), lung WT (n=6–13), lung ICOSL ^-/-^ (n=4–11), mLN WT (n=5–7) and mLN ICOSL ^-/-^ (n=5–7) are pooled from a minimum of 3 independent experiments for each tissue. Values on flow cytometry plots represent percentages, bars on scatter plots represents the median, which is also shown numerically for clarity. Statistical significance was tested using an unpaired, non-parametric, Mann-Whitney two tailed T test: *p≤0.05, **p≤0.01, ***p≤0.001.

Given the relatively recent identification of many ILC populations, it is unsurprising that our understanding of the role of many molecules expressed by ILC is limited. Interestingly, the initial characterisation of ILC2, alongside early descriptions of LTi-like cells, have identified ILC expression of costimulatory molecules with known roles in CD4 T cell responses ^3^. In our hands, ILC3 expression of ICOS in the SI LP was restricted to the CCR6 ^-^ fraction, with a homogenous level of expression comparable to that observed on ILC2 ( Figure 4A). Signalling through ICOS has been reported to increase ILC2 proliferation in both the lung and intestine, whilst ICOS ^-/-^ mice are described as having fewer ILC2 in the lung ^19, 20^. Employing our broad characterisation of tissue ILC populations, we sought to assess the importance of signals through ICOS in ILC homeostasis. Thus, ILCs were isolated from the SI LP, lung and mLN of WT and ICOSL ^-/-^ mice and the total number of ILC as well as the proportion and total number of specific ILC groups assessed ( Figure 4B–D). In contrast to earlier reports defining ILC populations with cell surface markers alone, no differences in the total number of the ILC2 or ILC3 were observed in any tissue from WT and ICOSL ^-/-^ mice, indicating that ICOSL signals are not essential for ILC homeostasis in these tissues. Similarly, no change in the total number of ILC2 or ILC3 was evident when ILCs were isolated from the SI LP of ICOS ^-/-^ mice compared with WT ILCs, although modest differences in the number of ILC3 in mLN were observed ( Supplementary Figure 8). Although only CCR6 ^-^ ILC3 in the SI LP uniformly express ICOS, no differences were detected in the total number of different ILC3 subsets in ICOSL ^-/-^ SI LP ( Figure 4E) or mLN ( Figure 4F), arguing that CCR6 ^- ^ILC3 populations are not ICOSL dependent.

### ILC populations in mice lacking activated T cells

**Figure 5.**
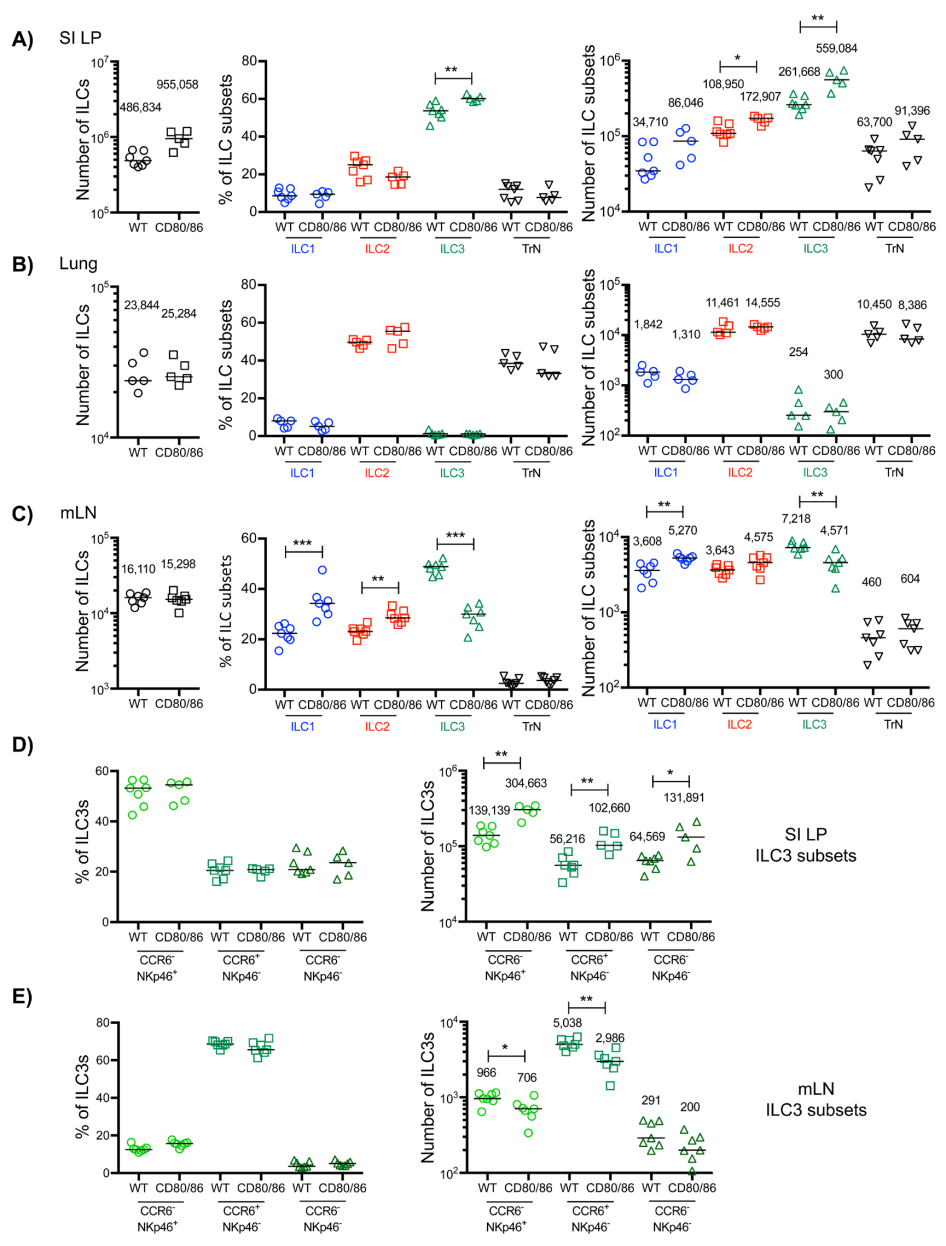
Mice deficient in CD80 and CD86 have increased ILC2 and ILC3 populations in the small intestine. To investigate the effect of blocking T cell activation on ILC populations, cells were prepared from the SI LP, lung and mLN of WT and CD80 ^-/-^CD86 ^-/-^ mice and ILC populations enumerated. ILCs were identified as live CD45 ^+^IL-7Rα ^+^Lin (CD3, CD5, B220, CD11b, CD11c) ^-^iCD3 ^-^ cells, transcription factor expression was used to identify ILC1 (T-bet ^+^, blue), ILC2 (GATA-3 ^+^, red), ILC3 (RORγt ^+^, green) and ‘Triple Negative’ cells lacking expression of the GATA-3, RORγt and T-bet (black). **A**– **C**) The total number of ILCs (left hand graph), the percentage of the ILCs within each group (centre graph) and the total number of each ILC group isolated from the SI LP ( **A**), lung ( **B**) and mLN ( **C**). **D**)– **E**) The percentage (left hand graph) and total number (right hand graph) of each ILC3 subset isolated from the SI LP ( **E**) and mLN ( **F**) of WT and CD80 ^-/-^CD86 ^-/-^ mice. Each data point represents cells isolated from 1 x SI LP, 1 x lung and 1 pool of mLNs isolated from one mouse. The data shown for SI LP WT (n=7), SI LP CD80 ^-/-^CD86 ^-/-^ (n=5), lung WT (n=5), lung CD80 ^-/-^CD86 ^-/-^ (n=5), mLN WT (n=7) and mLN CD80 ^-/-^CD86 ^-/-^ (n=7) are pooled from a minimum of 3 independent experiments for each tissue. Values on flow cytometry plots represent percentages, bars on scatter plots represents the median, which is also shown numerically. Statistical significance was tested using an unpaired, non-parametric, Mann-Whitney two tailed T test: *p≤0.05, **p≤0.01, ***p≤0.001.

Our analysis of ILC populations in mice deficient in ICOS or ICOSL indicated no clear effect on ILC populations despite some of the cells expressing ICOS expression. Whilst there is little clear evidence that ILC express the CD28 ligands CD80 and CD86, there is evidence to support ILC expression of CD28 ^25^. Furthermore, ILC cross talk with activated T cells has been suggested by several studies ^7, 14, 15^, arguing that disruption of T cell activation may impact upon ILC homeostasis. In addition, the cytokines that support ILC homeostasis, IL-7 and IL-15, are similarly required for T cell populations, and competition for these molecules has been proposed as a mechanism limiting ILC and T cell numbers ^16, 26^. Thus we considered that it would be informative to extend our analysis of ILC populations to mice deficient in both CD80 and CD86, where signals through CD28 are deficient and thus T cell activation is blocked ( Figure 5 A–C). No differences were observed within lung ILC populations of WT and CD80 ^-/-^86 ^-/-^ mice, however the ILC3 population in the SI LP was significantly enhanced ( Figure 5A), with expanded numbers of all ILC3 subsets ( Figure 5D). In addition, the ILC3 populations in the mLN were reduced in the absence of CD80 and CD86 signals. ( Figure 5C, E). These data suggest that ILC populations in some tissues are enhanced in the absence of T cell activation or perhaps that T cell activation can restrain ILC numbers.

## Discussion

In this study, we have used a flow cytometric approach based upon identification of transcription factor expression to characterise the ILC populations present within different murine tissues. This approach was taken to avoid confusion resulting from tissue specific variations in surface molecule expression and to provide a simple overview of the composition of ILC populations within mice from the same colony. Whilst our data is consistent with existing descriptions of ILCs in tissues such as the intestine, lung and mLN ^7, 10, 13^, a surprising observation that emerged was the identification of IL-7Rα ^+^Lin ^-^ cells lacking clear expression of the transcription factors used to define the ILC groups. The proportion of IL-7Rα ^+^Lin ^-^ cells with this phenotype differed markedly between tissues and was most noticeable within ear tissue which, to our knowledge has not been assessed with in this manner previously. The robust expression of the different transcription factors in samples analysed alongside the ear skin argues against an inability to detect expression of these transcription factors. Furthermore, we were unable to diminish this population through extending the cocktail of lineage defining Abs used in the study. Additionally, we provide evidence that these cells express Id2, a defining feature of ILCs. However, when we tested whether the manner in which we digest tissue can affect the proportion of different ILC populations, it is was evident that for lung tissue at least, using the same enzymatic mix for longer and at a higher concentration changed the ILC populations that were detectable. While a novel regulatory ILC population lacking expression of RORγt, GATA-3 and T-bet was very recently described ^27^, the weight of evidence presented here indicates that the RORγt ^-^GATA-3 ^-^T-bet ^-^ population we observe in the skin is at least partially influenced by the prolonged digestion and high enzyme concentration used to isolate the cells and likely includes ILCs that are losing transcription factor expression as a result of how they are isolated from the tissue. We demonstrate that by increasing the digestion of lung tissue, the frequency of GATA-3+ ILC2 isolated from the tissue is reduced and the proportion of ILC lacking expression of GATA-3, T-bet and RORγt is increased. Thus we conclude that whilst ILC lacking expression of the three group defining transcription factors may exist, care must be taken in interpreting data from digested tissue and this population can certainly be ‘contaminated’ by *ex vivo* changes to known ILC populations during their isolation and analysis. Whilst ILC progenitor populations within different tissues remain to be fully characterised, these observations highlight how the manner in which different tissues are digested can impact on our ability to properly identify different ILC populations.

Whilst we have tried to phenotype ILC populations isolated from different tissues, our data again highlights the potential caveats to studying phenotypic markers following substantial digestion of tissue. Whilst we could detect a robust ILC2 population in ear skin following the digestion protocol used, we are left unable to comment on the expression of CD25 and KLRG-1 at the protein level due to clear loss of these markers during cellular isolation. We do provide evidence that ILCs could still be clearly distinguished since expression of key lineage markers were not lost.

The tissue specific composition of ILC groups and also ILC3 subsets are consistent with many studies of ILCs, but are striking when total ILC groups in multiple tissues are viewed together. Having characterised these populations in WT mice, we sought to compare this cellular distribution with mice in which costimulatory molecules were deficient, focusing initially on ICOS, since in addition to its described expression by ILC2 ^3^, we detected ICOS protein expression on the surface of ILC3 in the SI LP, but only those lacking CCR6 expression. Surprisingly, we observed no clear defect in the total number of ILC populations in the different tissues analysed, in contrast to other studies ^19, 20^. Whilst this argues that this pathway is not essential for normal ILC homoeostasis, it is worth pointing out that this is analogous to the role of ICOS in T cell development, where ICOS is required for differentiation of effector T cells rather than normal naïve T cell development ^28^. Whilst these data conflict with previous reports on the requirement for ICOS-ICOSL in ILC2 homeostasis, this may reflect differences arising from the animal facility in which the studies were conducted. We decided to characterise ILC populations in CD80 ^-/-^CD86 ^-/-^ mice since there is evidence to support ILC expression of CD28 and a block in T cell activation might reveal further insight into the effects of ILC:T cell cross talk ^29– 31^. It was noticeable that in the intestine, the ILC3 population was enhanced, which might reflect greater availability of cytokines such as IL-7 and IL-15, although the absence of T cell responses to the commensal microbial population is obviously a further potential factor. Alternatively, T cell populations may constrain ILCs and the inability form activated T cell populations and regulatory T cells may permit ILC expansion in some tissues. Having only assessed ILC populations in total knockout mice, it is also impossible to really separate ILC-intrinsic effects arising from expression of CD28 or microenvironmental effects resulting from changes in T cell activation. To really understand these pathways, novel conditional knockout mice targeting these costimulatory pathways in ILC versus T cells need to be developed.

## Conclusions

 In this study we have tried to broadly, but definitively assess ILC populations in a range of tissues. While using this analysis in mice is a fairly blunt tool for understanding ILC biology, it does provide some initial experimental observations as to ILC requirements *in vivo*. ILCs express an array of different costimulatory receptors and ligands, yet we know very little about how many of these molecules contribute to ILC function. As we look to understand the roles of ILCs within an intact immune system, this is an important and growing area of research, particularly given the wealth of therapeutics that target these pathways.

## Data availability

Data are stored on the online depository, Open Science Framework: http://doi.org/10.17605/OSF.IO/WGZAM ^32^.

Data are available under the terms of the Creative Commons Zero “No rights reserved” data waiver (CC0 1.0 Public domain dedication).

## Supplementary material

Supplementary File 1: Supplementary Figure 1– Supplementary Figure 8.

**Supplementary Figure 1. IL-7Rα ^+^Lineage ^-^ cells in ear skin.**

To better characterise the IL-7Rα ^+^Lineage ^-^ population identified in ear skin, cells were isolated from the tissue and stained with an extended panel of lineage specific markers (comprising B220, CD11b, CD11c, CD5, CD3, CD19, Ter119, CD123, Gr1, F4/80, Fc *ε*R1 and CD49b) and the population assessed by flow cytometry. **A**) Representative flow cytometry plots showing live CD45 ^+^IL-7Rα ^+^Lin-iCD3 ^-^ cells, with coloured gates identifying ILC1 (T-bet ^+^, blue), ILC2 (GATA-3 ^+^, red), ILC3 (RORγt ^+^, green) and ‘Triple Negative’ cells lacking expression of the GATA-3, RORγt and T-bet, black). **B**) Pie chart showing the mean proportion of each ILC group using the group-specific colours used in part ‘A)’. Cells falling outside of the transcription factor gates were termed ‘ungated’ and included to allow analysis of the entire ILC compartment. Data shown for 2x ears (n=8), values on flow cytometry plots represent percentages.

**Supplementary Figure 2. IL-7Rα ^+^Lineage ^-^ cells in ear skin express Id2.**

To provide further evidence that the IL-7Rα ^+^Lineage ^-^ lacking Gata-3, RORγt and T-bet resembled an ILC population, the IL-7Rα ^+^Lineage ^-^ population in the ear skin of Id2-eGFP reporter mice was assessed. **A**) Representative flow cytometry plots showing gating on live CD45 ^+^IL-7Rα ^+^Lin ^-^iCD3 ^-^ cells prepared from ear skin and auLN of Id2-eGFP mice versus WT controls (to aid gating on eGFP expression, shown in red). **B**) The proportion of eGFP ^+^ ILC (live CD45 ^+^IL-7Rα ^+^Lin ^-^iCD3 ^-^ cells) isolated from ear skin and auLN of Id2-eGFP mice. Data shown for 2x ears (n=5) and 2x auLN (n=5) pooled from two independent experiments.

Values on flow cytometry plots represent percentages; bars on scatter plots represent the median.

**Supplementary Figure 3. ILC transcription factor expression is reduced under the ear digestion protocol.**

To further evaluate the effect of the ear digestion protocol (EDP) on identifying ILCs within the ear, cells were isolated from the ear, using normal EDP, the lung using normal lung digestion protocol (LDP) and compared to cells isolated from the lung using EDP. ILCs were identified as live CD45 ^+^IL-7Rα ^+^Lin (CD3, CD5, B220, CD11b, CD11c, CD19, Ter119, Gr1, CD123, F4/80, CD49b, Fc *ε*RI) ^-^iCD3 ^-^ cells. **A**– **C**) Representative flow cytometry plots showing gating on ILCs (live CD45 ^+^IL-7Rα ^+^Lin ^-^iCD3 ^-^), ILC1s (T-bet ^+^), ILC2s (GATA-3 ^+^), ILC3s (RORγt ^+^) and TrN (RORγt ^-^GATA-3 ^-^T-bet ^-^) cells within the ear using EDP, lung using LDP and lung using LDP. **D**) The percentage (left hand graph) and total number (right hand graph) of live CD45 ^+^ cells within the lung under EDP (n=6) or LDP (n=6). **E**) The percentage (left hand graph) and total number (right hand graph) of ILC within the lung using LDP or EDP. **F**) The percentage (left hand graph) and total number (right hand graph) of ILC subsets within the lung using LDP or EDP. Data are from two experiments, values of flow cytometry plots represent percentages, bars on scatter plots represents the median, which is also shown numerically for clarity. Statistical significance was tested using an unpaired, non-parametric, Mann-Whitney two tailed T test: *p≤0.05,**p≤0.01, ***p≤0.001.

**Supplementary Figure 4. Surface markers used to identify ILC are not affected by the ear digestion protocol.**

To evaluate whether cell surface markers identified by Abs in the lineage cocktail were affected by the EDP, cells were isolated from the ear, using normal EDP, the lung using normal lung digestion protocol (LDP) and compared to cells isolated from the lung using EDP. **A**) Representative flow cytometry plots showing expression of the cell surface markers CD3 *ε*, CD5, CD49b, F4/80 and Fc *ε*RI on live CD45 ^+^ cells isolated from the ear using EDP, lung using LDP and lung using LDP. **B**) Percentage of live CD45 ^+^ cells expressing the cell surface markers CD3 *ε*, CD5, CD49b, F4/80 and Fc *ε*RI within the lung using LDP (n=6) and lung using EDP (n=6). Data are from two experiments, values of flow cytometry plots represent percentages, bars on scatter lots represents the median. Statistical significance was tested using an unpaired, non-parametric, Mann-Whitney two tailed T test: *p≤0.05,**p≤0.01, ***p≤0.001.

**Supplementary Figure 5. Specific transcription factors and surface markers used characterise ILC subsets are affected by the ear digestion protocol.**

To evaluate whether transcription factors and other cell surface markers used to identify ILC subgroups were affected by the EDP, cells were isolated from the ear, using normal EDP, the lung using normal lung digestion protocol (LDP) and compared to cells isolated from the lung using EDP. **A**) Representative flow cytometry plots showing expression of transcription factors RORγt, Tbet and GATA-3 and expression of the cell surface markers CD4 and CD8 on live CD45+ cells isolated from the ear using EDP, lung using LDP and lung using LDP. **B**) Percentage of live CD45 +cells expressing the transcription factors RORγt, Tbet and GATA-3 and the cell surface markers CD4 and CD8 within the lung using EDP (n=6) and lung using LDP (n=6). Data are from two experiments, values of flow cytometry plots represent percentages, bars on scatter plots represent the median. Statistical significance was tested using an unpaired, non-parametric, Mann-Whitney two tailed T test: *p≤0.05,**p≤0.01, ***p≤0.001.

**Supplementary Figure 6. Specific surface markers used characterise ILC2s are affected by the ear digestion protocol.**

To evaluate whether cell surface markers used characterise ILC2s were affected by the EDP, cells were isolated from the ear, using normal EDP, the lung using normal lung digestion protocol (LDP) and compared to cells isolated from the lung using EDP. **A**) Representative flow cytometry plots showing expression the cell surface markers CD25, ICOS, KLRG1, MHCII and ST2 on live CD45 ^+^ cells isolated from the ear using EDP, lung using LDP and lung using LDP. **B**) Percentage of live CD45 ^+^ cells expressing the cell surface markers CD25, ICOS, KLRG1, MHCII and ST2 within the lung using EDP (n=6) and lung using LDP (n=6). Data are from two experiments, values of flow cytometry plots represent percentages, bars on scatter plots represent the median. Statistical significance was tested using an unpaired, non-parametric, Mann-Whitney two tailed T test: *p≤0.05,**p≤0.01, ***p≤0.001.

**Supplementary Figure 7. Characterisation of ILC2s surface phenotype through flow cytometry in different tissues.**

To further characterise the ILC2 population identified in the different tissues, expression of CD25, ICOS, KLRG-1, MHCII and ST2 was assessed. **A**) Representative flow cytometry plots showing expression of the cell surface markers CD25, ICOS, KLRG-1, MHCII and ST2 on GATA-3 ^+^ILCs within the ear, SI, lung and mLN. **B**) Histograms showing expression of each marker by either GATA-3 ^+^ILC2 or Lineage ^+^IL-7Rα ^-^ cells, which were used to help with gating. Values of flow cytometry plots represent percentages.

**Supplementary Figure 8. Numbers of ILC2 and ILC3 in ICOS ^-/-^ mice.**

To further assess the role of ICOS:ICOSL interactions in ILC homeostasis, cells were isolated from the SI LP and mLNs of WT and ICOS ^-/-^ mice and ILC groups enumerated. ILCs were identified as live CD45 ^+^IL-7Rα ^+^Lin (CD3, CD5, B220, CD11b, CD11c) ^-^iCD3 ^-^ cells, transcription factor expression was used to identify ILC1 (T-bet ^+^, blue), ILC2 (GATA-3 ^+^, red), ILC3 (RORγt ^+^, green) and ‘Triple Negative’ cells lacking expression of the GATA-3, RORγt and T-bet (black). **A**– **B**) The total number of ILCs (left hand graph), the percentage of the ILCs within each group (centre graph) and the total number of each ILC group isolated from the SI LP ( **A**), and mLN ( **B**). Each data point represents cells isolated from 1× SI LP and 1 pool of mLNs isolated from one mouse. The data shown for SI LP WT and ICOS ^-/-^ (n = 10) and mLN WT and ICOS ^-/-^ (n = 10) are pooled from 3 independent experiments. Bars on scatter plots represent the median, which is also shown numerically. Statistical significance was tested using an unpaired, non-parametric, Mann-Whitney two tailed T test: *p≤0.05,**p≤0.01, ***p≤0.001.
