# Supplementary material for: Characterisation of innate lymphoid cell populations at different sites in mice with defective T cell immunity
Source: Wellcome Open Res. 2018 Mar 14;2:117. Originally published 2017 Dec 14. [Version 3] doi: 10.12688/wellcomeopenres.13199.3 (PMC5854988; doi:10.12688/wellcomeopenres.13199.3)
Supplement: Supplementary file 1 [file wellcomeopenres-2-15451-s0000.tgz › 7da11f14-1851-4bc6-84b0-620230774ae2.pdf]

A)

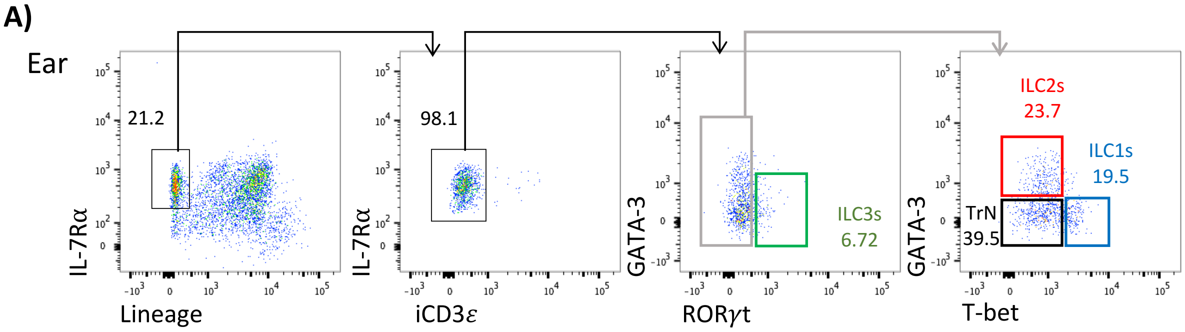

B)

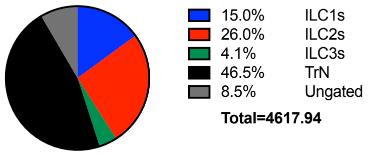

Lineage Gate:
B220, CD11b, CD11c, CD5, CD3, CD19, Ter119, CD123, Gr1, F4/80, FcεR1 and CD49b

Supplementary Fig1

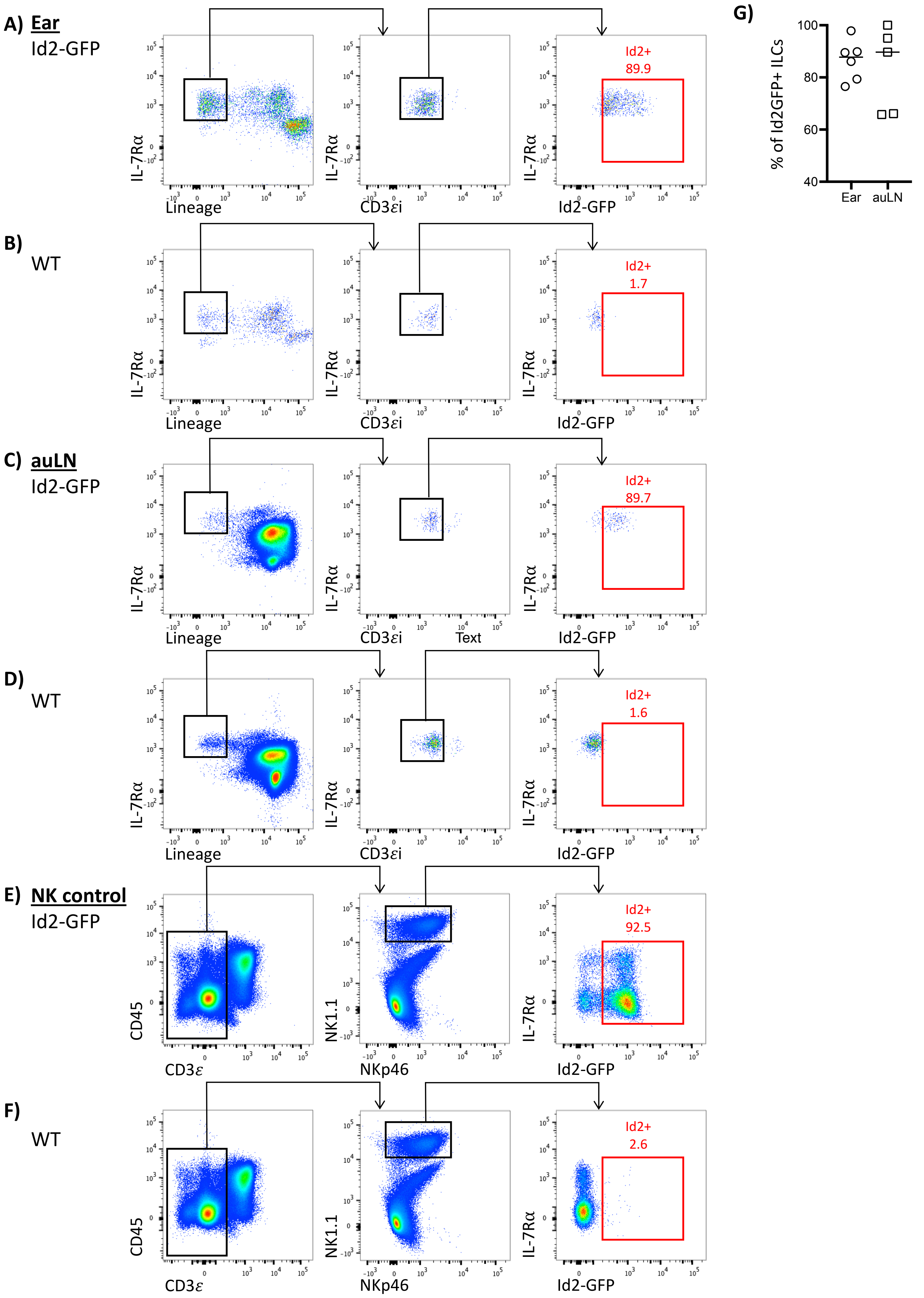

Supplementary Fig2

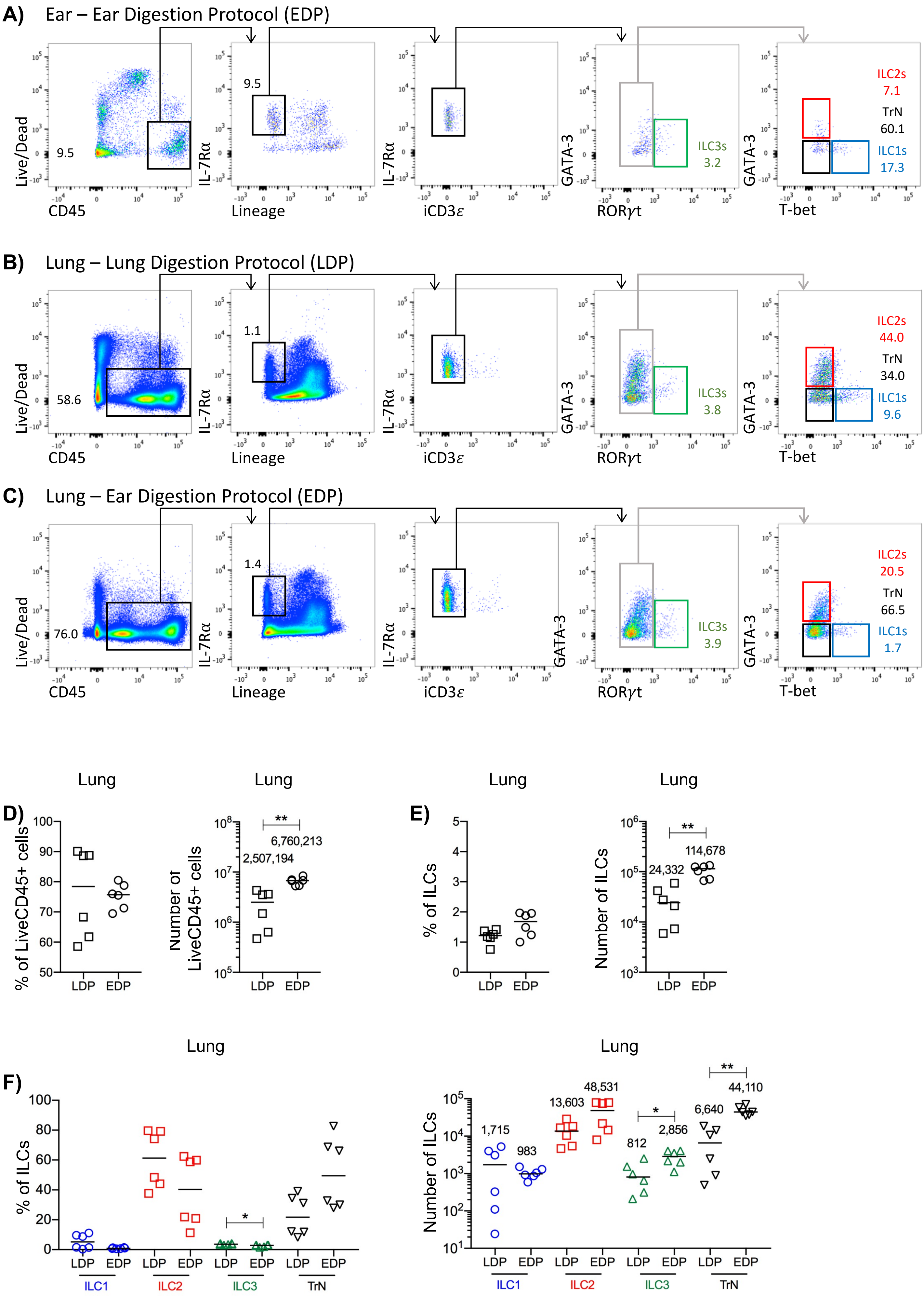

Supplementary Fig3

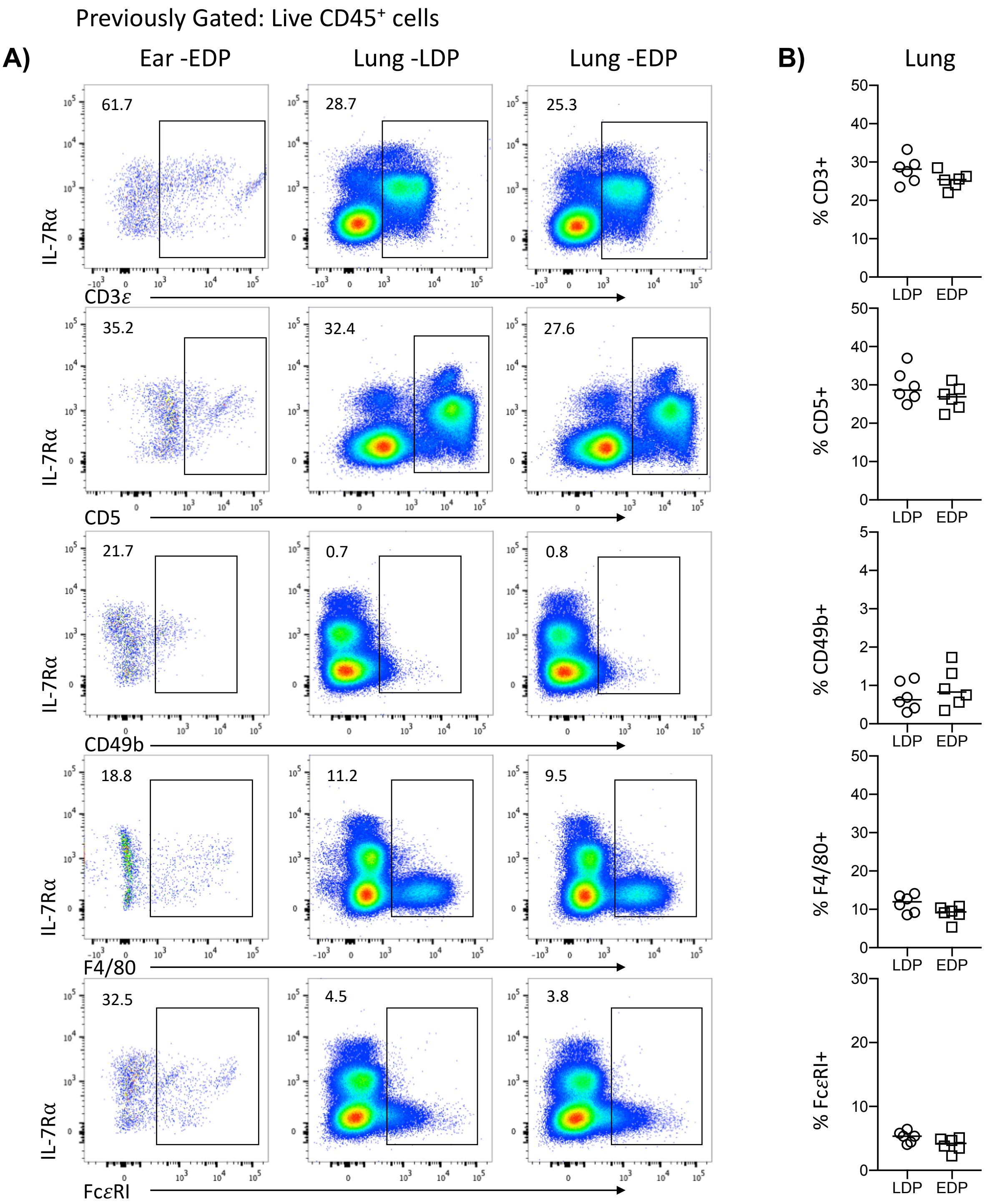

Supplementary Fig4

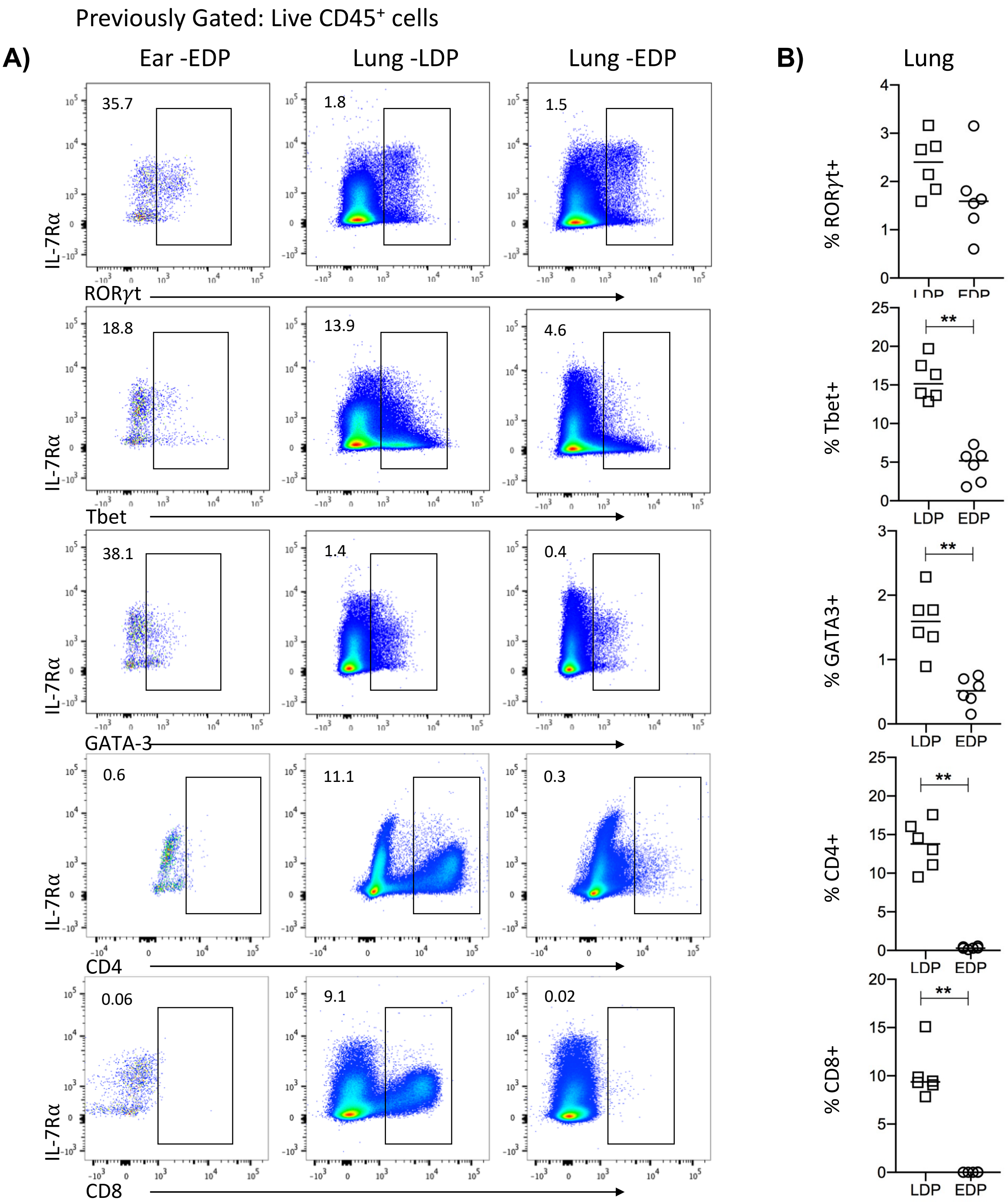

Supplementary Fig5

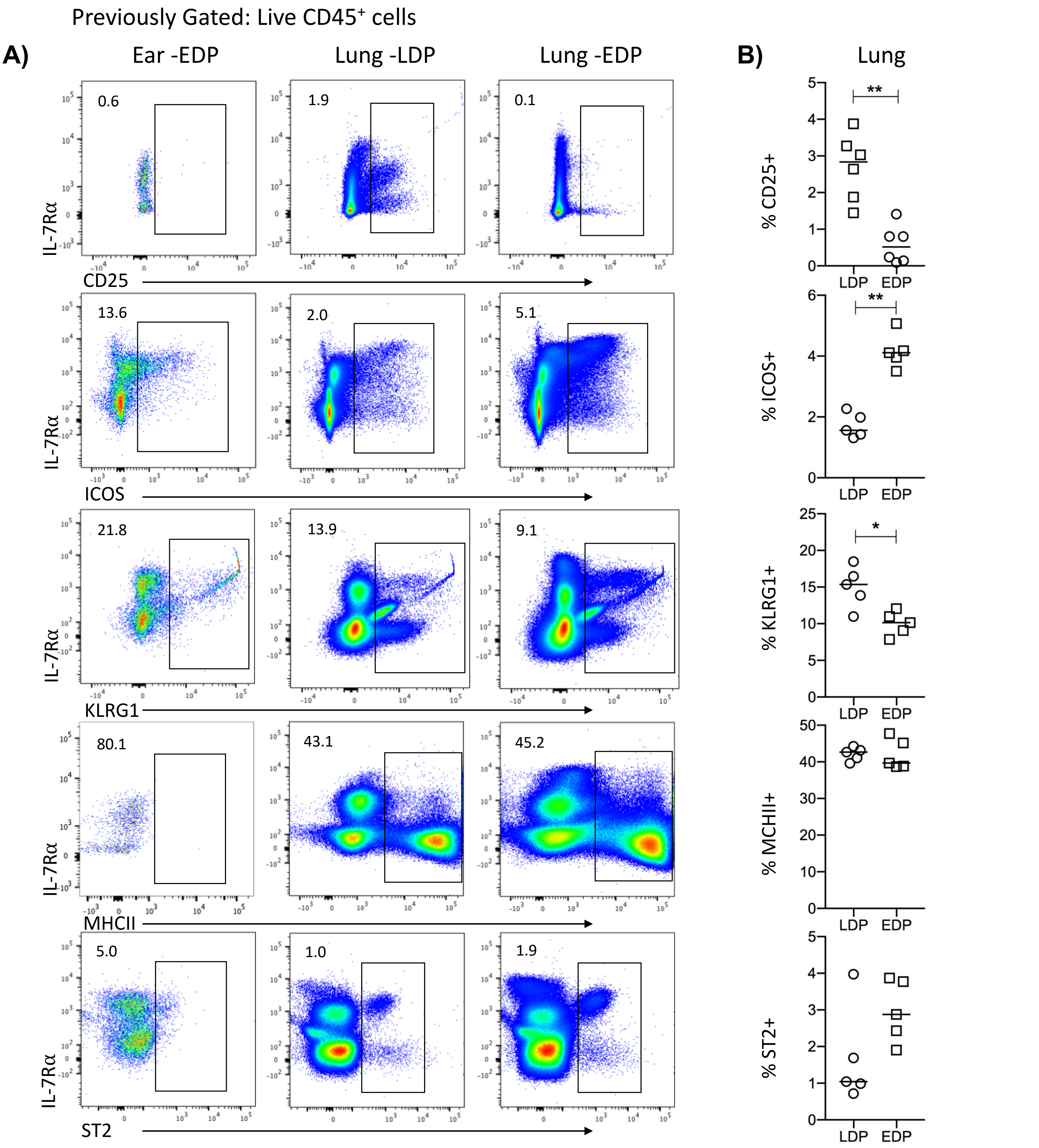

Supplementary Fig6

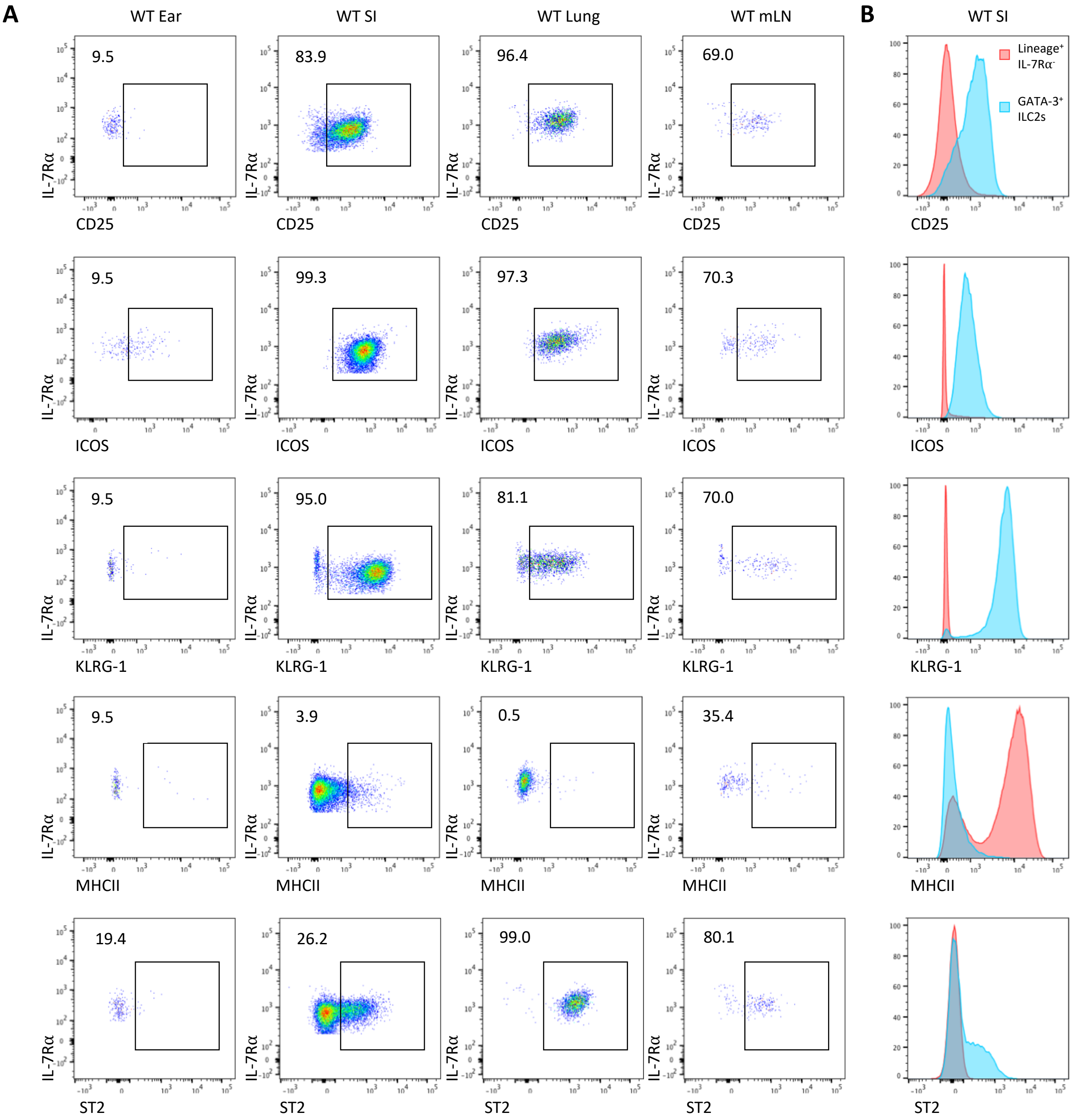

Supplementary Fig7

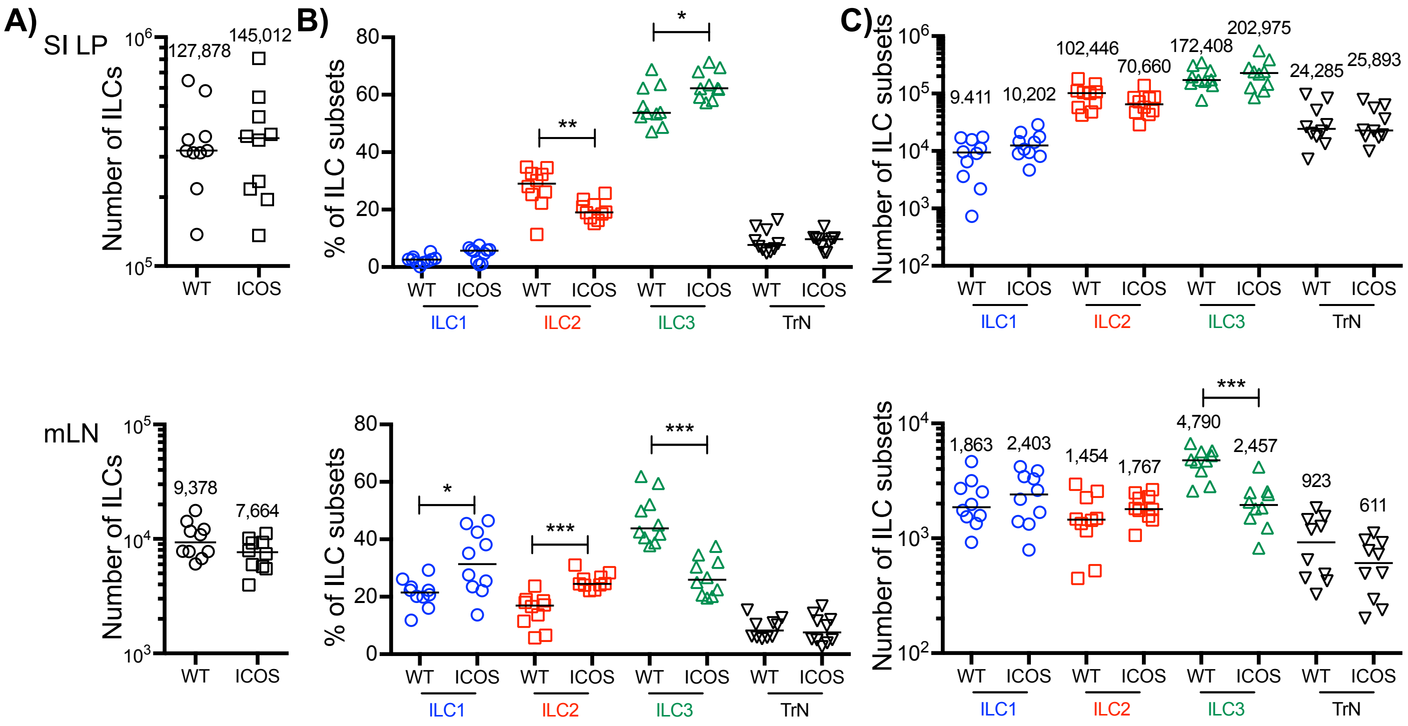

Supplementary Fig8
