# Supplementary material for: Characterisation of innate lymphoid cell populations at different sites in mice with defective T cell immunity
Source: Wellcome Open Res. 2018 Mar 14;2:117. Originally published 2017 Dec 14. [Version 3] doi: 10.12688/wellcomeopenres.13199.3 (PMC5854988; doi:10.12688/wellcomeopenres.13199.3)
Supplement: Peer review history

# Peer review: Referee response for version 3

**DOI:** 10.21956/wellcomeopenres.15451.r31902

**Published:** 2018-03-15

**Authors:** Nick Powell

**Affiliations:** 1 School of Immunology & Microbial Sciences, King's College London, London, UK

I am happy with the changes made and think that this is an important contribution to the field.

I have read this submission. I believe that I have an appropriate level of expertise to confirm that it is of an acceptable scientific standard.

# Peer review: Referee response for version 2

**DOI:** 10.21956/wellcomeopenres.14825.r29454

**Published:** 2018-01-17

**Authors:** Nick Powell

**Affiliations:** 1 School of Immunology & Microbial Sciences, King's College London, London, UK

The paper addresses 4 issues.

*1) A descriptive comparison of the relative and absolute ILC subset composition in some tissues of adult B6 mice in the steady state.*

*2) Identifies that different tissue digestion protocols employed to isolate immune cell fractions may be linked to loss of particular lineage defining markers in ILCs (and other immune cells)*

*3) An analysis of the impact of ICOSL deficiency on the composition of ILCs in various tissues*

*4) An analysis of the impact of CD80/86 deficiency on the composition of ILCs in various tissues*

**General comments**

 As well as providing a useful "atlas" of ILC subset distribution in different tissues in the steady state, the study also highlights the presence of a population of cells, that are deemed ILCs (defined by absence of major lineage markers and IL7 ^+^), that lack expression of any lineage defining transcription factors (RORgt, GATA3, T-bet). The authors term these cells triple negative (TrN) ILCs.

 The paper also nicely demonstrates a potentially important technical issue in the ILC field, in that tissue digestion protocols (most importantly dose and duration of enzyme exposure) seems to significantly impact ILC composition using conventional gating strategies. Although it is easy to envisage how high dose enzymes might shave off some surface antigens, it is more conceptually challenging to understand how tissue processing impacts intracellular transcription factor expression. Another possibility is that enzyme treatment leads to loss of lineage defining antigens, resulting in lineage positive cells erroneously appearing in the lineage negative gate (and hence potentially contributing to the variable ILC and TrN ILC pool). 

 Notwithstanding that a separate technical paper about isolation protocols and ILC marker stability might be needed, I would be intrigued to see how the different digestion protocols impact the expression of all ILC markers in tissue where these cells can be isolated without enzymatic digestion (e.g. splenic ILCs can be visualized by flow cytometry by mechanical dissociation without the need for enzymatic digestion), allowing systematic analysis of ILC marker changes without digestion vs all other digestion protocols.

**Specific comments**

 The manuscript could be improved if the following issues were addressed.

 1) Regulatory ILCs should now be considered within the extended ILC family, and given that the presence of "lineage undefined" ILCs in different tissues is a major finding of the paper, there would be merit in more in depth phenotyping of the TF negative population. Unsupervised transcriptomics (e.g. RNA-seq) would provide an unbiased assessment of the cellular identity these cells. However, given the potential relevance of ILCregs, an alternative approach would be to consider performing qPCR to evaluate ILCreg molecules (e.g. Il10, Sox4, etc) in the TF negative population.

 2) I would question the value of the ID2 reporter experiments. ID2 is expressed by a wide variety of lymphocytes (and certainly not just by ILCs), and therefore, I'm not sure it helps convince me that the unknown TF negative cells are ILCs, or even narrow down the search (beyond lymphocytes).

 3) I would be intrigued to see how the composition of ILC subsets differs in the different digestion protocols using TF reporter mice (rather than intracellular TF staining), as these data raise some important considerations for the field, and strategies to mitigate against this problem are now needed. This is probably be beyond the remit of the current paper, but would be a useful "technical report" to help shape future experiments in the field

 4) I would like to see representative flow plots of MHCII, CD25 etc (Figure 3) as a supplemental figure.

 5) In this study there was no impact on the composition of tissue ILC populations in mice with germ line genetic deletion of ICOSL. This is in variance to other published reports that have shown significant reductions in lung ILC2 in mice with disruption of the ICOS/ICOSL axis (albeit with ICOS deficient animals). Although in previous published work this tends to be most apparent in IL33 treated animals - it is also clearly apparent in the steady state (60-70% reduction in lung ILC2 in *Icos ^-/-^* animals). The manuscript would benefit from a more in depth discussion reconciling this discrepancy.

 6) The interpretation of the *Cd80 ^-/-^ x Cd86 ^-/-^* experiments is probably overstated. I would be cautious of attributing the ILC phenotype observed (expansion of intestinal ILC2 and ILC3) to unactivated T-cells, especially as ILCs may express CD28 (as well as checkpoint molecules, PD-1, which modulate CD28 signalling). Further experiments are needed to conclusively demonstrate whether the observed shift in ILC composition in *Cd80 ^-/-^ x Cd86 ^-/- ^*animals is due to a direct effect of loss of CD80/86 interaction with ILCs, or as the authors imply,  by loss of T-cell activation. The authors could address this by "relaxing" the interpretation of their findings and by entertaining some alternative possibilities (as described above). This would potentially include changing the title of the manuscript.

 In short, the m/s brings important new information to the table. A few minor (mostly editorial) changes would improve the article.

I have read this submission. I believe that I have an appropriate level of expertise to confirm that it is of an acceptable scientific standard, however I have significant reservations, as outlined above.

# Response

**Published:** 2018-03-06

**Authors:** Emma Dutton

**Affiliations:** University of Birmingham, UK, UK

We thank the reviewer for the constructive criticism and have responded to the specific comments below:

Question 1: Regulatory ILCs should now be considered within the extended ILC family and given that the presence of "lineage undefined" ILCs in different tissues is a major finding of the paper, there would be merit in more in depth phenotyping of the TF negative population. Unsupervised transcriptomics (e.g. RNA-seq) would provide an unbiased assessment of the cellular identity these cells. However, given the potential relevance of ILCregs, an alternative approach would be to consider performing qPCR to evaluate ILCreg molecules (e.g. Il10, Sox4, etc) in the TF negative population.

*Answer 1: We thank the reviewer for this suggestion. Whilst we have considered a more in depth assessment of the TrN population which could be described as resembling a putative regILC cell, the fact that the detection of these cells was very dependent upon the digestion protocol performed greatly reduced our enthusiasm to study these cells further. We are concerned that the ‘novel’ population described here is artefactual and reflects a loss of transcription factor expression under harsh isolation conditions. The fact that changing the digestion of the lung tissue greatly affects the detection of these cells (alongside the conventional ILC groups) really argues that these cells are unlikely to be a real biologically relevant population. If regILC really do exist then we would agree that further more in depth experiments from other groups would substantiate this potentially new field.*

Question 2: I would question the value of the ID2 reporter experiments. ID2 is expressed by a wide variety of lymphocytes (and certainly not just by ILCs), and therefore, I'm not sure it helps convince me that the unknown TF negative cells are ILCs, or even narrow down the search (beyond lymphocytes).

*Answer 2: As mentioned in response to a similar comment from Reviewer 1, we agree that Id2 is not a definitive ILC marker, but having gated out lineage expressing cells, detection of Id2 expression by the majority of the IL-7Rα+ Lin- population was supportive of the cells being ILC-like. The aim of this experiment was to ask whether the TrN population, which was >50% of the IL-7Rα+ Lin- population in the ear, expressed Id2 and thus might be considered to be ILC-like in this regard. This was the only point we were trying to make with the Id2-reporters and we have tried to amend the text to make this clearer.*

Question 3: I would be intrigued to see how the composition of ILC subsets differs in the different digestion protocols using TF reporter mice (rather than intracellular TF staining), as these data raise some important considerations for the field, and strategies to mitigate against this problem are now needed. This is probably be beyond the remit of the current paper, but would be a useful "technical report" to help shape future experiments in the field

Answer 3: We agree this would be an interesting experiment to perform but we do not have access to the relevant mouse models.

Question 4: I would like to see representative flow plots of MHCII, CD25 etc (Figure 3) as a supplementary figure.

*Answer 4: We agree with the reviewer that this should have been shown and these data have now been added to the revised supplementary figures (Supplementary Figure 7).*

Question 5: In this study there was no impact on the composition of tissue ILC populations in mice with germ line genetic deletion of ICOSL. This is in variance to other published reports that have shown significant reductions in lung ILC2 in mice with disruption of the ICOS/ICOSL axis (albeit with ICOS deficient animals). Although in previous published work this tends to be most apparent in IL33 treated animals - it is also clearly apparent in the steady state (60-70% reduction in lung ILC2 in *Icos ^-/-^* animals). The manuscript would benefit from a more in depth discussion reconciling this discrepancy.

*Answer 5: We thank the reviewer for this comment and have tried to discuss this point further. We were surprised that in our hands we failed to observe differences in ILC2 numbers in neither ICOS-/- nor ICOSL-/- mice versus controls, given the other reports. Having repeated the experiments a number of times we felt that whilst there was no reproducible difference in our hands this was clearly distinct from the findings of others for reasons unclear. It seems likely that ILC numbers will vary between different animal facilities and this may be influencing our findings. We have inserted the following sentence:*

*‘Whilst these data conflict with previous reports on the requirement for ICOS-ICOSL in ILC2 homeostasis, this may reflect differences arising from the animal facility in which the studies were conducted.’*

Question 6: The interpretation of the *Cd80 ^-/-^ x Cd86 ^-/-^* experiments is probably overstated. I would be cautious of attributing the ILC phenotype observed (expansion of intestinal ILC2 and ILC3) to unactivated T-cells, especially as ILCs may express CD28 (as well as checkpoint molecules, PD-1, which modulate CD28 signalling). Further experiments are needed to conclusively demonstrate whether the observed shift in ILC composition in *Cd80 ^-/-^ x Cd86 ^-/- ^*animals is due to a direct effect of loss of CD80/86 interaction with ILCs, or as the authors imply, by loss of T-cell activation. The authors could address this by "relaxing" the interpretation of their findings and by entertaining some alternative possibilities (as described above). This would potentially include changing the title of the manuscript.

*Answer 6: We agree with the reviewer and have modified our discussion here and included the following text:*

*‘Having only assessed ILC populations in total knockout mice, it is also impossible to really separate ILC-intrinsic effects arising from expression of CD28 or microenvironmental effects resulting from changes in T cell activation. To really understand these pathways, novel conditional knockout mice targeting these costimulatory pathways in ILC versus T cells need to be developed.’*

# Peer review: Referee response for version 2

**DOI:** 10.21956/wellcomeopenres.14825.r29042

**Published:** 2018-01-08

**Authors:** Amy E. Saunders

**Affiliations:** 1 Manchester Collaborative Centre for Inflammation Research, University of Manchester, Manchester, UK

This article provides a detailed comparison of ILC subsets in different tissues including ear skin, lamina propria, lung and various lymph nodes, including analysis of NKp46 and CCR6 expressing subsets of ILC3.  

 This analysis identified a subset of ILCs that do not express any of the lineage defining transcription factors (TrN cells), which are particularly abundant in skin and lung. The authors have made efforts to show that these cells are ILCs, and their analysis of different digestion protocols suggests that these cells may have lost expression of transcription factors due to the tissue digest procedure. This is an important consideration for other research examining ILCs in these tissues.

 The authors also examine the effects of the costimulatory molecules ICOSL and CD80/86 on the subsets of ILCs present in tissues. They show conclusively that the absence of ICOSL does not have major effects on the ILCs present, however the absence of CD80/86 causes an increase in ILC2 and ILC3 populations in the small intestine. It is unclear if the later finding is due to the lack of CD80/86- CD28 interactions on ILCs themselves, or if it is due to the absence of activated T cells.

 To improve the clarity of the article I suggest the following changes:

  

1. The materials and methods could be written more clearly in places. In particular, there is a reference to spleen digestions however, there doesn’t appear to be any data from spleen included in the manuscript. It is unclear if collagenase, or dispase, or both were used to digest lymph nodes and the centrifugation performed is described in rpm, but it is more useful for the reader to quote the force used. For lung and ear digests the final concentrations of enzymes should be clearly stated, as it is currently unclear if the concentrations given are stock concentrations or the final concentrations used in the digests.

 

 I also suggest that the following additions could be made to enhance the article:

  

1. The data shown here suggest that prolonged tissue digestion, or a high concentration of enzyme used to digest the tissue, leads to a higher proportion of the ILCs being TrN cells, which is likely due to a reduction in transcription factor expression. It would be interesting to ascertain if this is due to the activity of the enzymes directly affecting the transcription factor expression, or if it is due to prolonged incubation at 37 degrees. and this additional information may aid protocol optimization.
2. The authors show that the majority of the CD127 positive, lineage negative cells are Id2 positive, which suggests that these cells are ILCs. However, despite Id2 being required for ILC differentiation, it is not a specific marker for ILCs as other immune cells have been shown to express this transcriptional regulator, and these data are therefore not conclusive, but are suggestive. An approach to more definitively analyse the TrN population would be to perform RNAseq analysis and compare the findings to known immune cell subsets, however this may be outside of the scope of this paper.    
3. It would be interesting to digest the lung with the ear digest protocol and then analyse the cell surface markers examined in Figure 3 (CD25, ICOS, KLRG-1, MHCII and ST2), to ascertain if the digest protocol used affects the expression of these markers, particularly given the large differences seen in expression of these markers between lung and ear skin. 
4. The expression of markers such as CD25, KLRG1 and ST2 are shown in this report to be expressed at different levels on ILC2s in skin versus ILC2s in lung and gut. The potential reasons behind this differential expression could be addressed more fully in the discussion.
5. The data showing increased populations of ILC2 and ILC3 in CD80/86 deficient mouse gut, but lower numbers of ILC3s in mesenteric lymph nodes is intriguing, but it is unclear if this is caused by the lack of CD80/86 signals acting directly on ILCs, or if it is due to a lack of activated T cells in the absence of CD80/86. It would therefore be interesting to analyse ILCs in other transgenic mice where T cell activation is impaired to determine if ILC populations are affected by the presence of activated CD4 or CD8 T cells. Given that the ILC3 population is increased in the lamina propria and decreased in the mesenteric lymph node, it would also be interesting to determine if the increase in these ILC populations in the lamina propria is due to increased turnover, or increased trafficking of these cells or their precursors.

Overall this article makes important contributions to the field by describing the ILC subsets in different tissues, highlighting the TrN ILC cell population and demonstrating that the costimulatory molecule ICOSL does not affect ILC numbers, whereas CD80/86 does affect ILC subsets in the gut and mesenteric lymph nodes.  

I have read this submission. I believe that I have an appropriate level of expertise to confirm that it is of an acceptable scientific standard.

# Response

**Published:** 2018-03-06

**Authors:** Emma Dutton

**Affiliations:** University of Birmingham, UK, UK

We thank the reviewer for the constructive criticism and have responded to the specific comments below:

 Comment 1: The materials and methods could be written more clearly in places. In particular, there is a reference to spleen digestions however, there doesn’t appear to be any data from spleen included in the manuscript. It is unclear if collagenase, or dispase, or both were used to digest lymph nodes and the centrifugation performed is described in rpm, but it is more useful for the reader to quote the force used. For lung and ear digests the final concentrations of enzymes should be clearly stated, as it is currently unclear if the concentrations given are stock concentrations or the final concentrations used in the digests.

*Answer 1: We thank the reviewer for spotting this and we have amended the text in the methods in an effort to more clearly describe what was done. We have omitted the description of spleen cell preparation as this was not used in the data shown.*

 Comment 2: The data shown here suggest that prolonged tissue digestion, or a high concentration of enzyme used to digest the tissue, leads to a higher proportion of the ILCs being TrN cells, which is likely due to a reduction in transcription factor expression. It would be interesting to ascertain if this is due to the activity of the enzymes directly affecting the transcription factor expression, or if it is due to prolonged incubation at 37 degrees. and this additional information may aid protocol optimization.

*Answer 2: We agree with the reviewer that the prolonged tissue digestion seems to reduce expression of the transcription factors. We have provided further data in the revised supplementary figures showing expression of the transcription factors and different surface markers with different digestions of lung tissue. These data indicate that loss of T-bet and Gata-3 expression occur with the prolonged digestion protocol used to isolate cells from the ear. Based on expression of the transcription factors after ex vivo stimulation in other experiments (e.g. PMA/IONO), we think that the loss of expression is not simply a result of culture at 37 degrees and reflects the presence of high concentrations of enzyme. It may be that the harsh conditions in the culture result in some of the cells down regulating these transcription factors rather than a direct effect of the enzymes (which is conceptually easier to consider where surface markers are expressed). The key point that we felt we needed to make is that ILC appearing to lack the three ‘signature’ transcription factors could be enhanced in number through different digestions demonstrating an artificial element to their detection and questioning their relevance in vivo.*

 Question 3: The authors show that the majority of the CD127 positive, lineage negative cells are Id2 positive, which suggests that these cells are ILCs. However, despite Id2 being required for ILC differentiation, it is not a specific marker for ILCs as other immune cells have been shown to express this transcriptional regulator, and these data are therefore not conclusive, but are suggestive. An approach to more definitively analyse the TrN population would be to perform RNAseq analysis and compare the findings to known immune cell subsets, however this may be outside of the scope of this paper.

*Answer 3: We agree that Id2 is not a specific marker for ILC and is widely expressed in activated T cells. BUT, in this data we had first gated on IL-7Rα+ Lineage- cells and then asked whether Id2 was expressed within this population in the ear where many ‘TrN’ cells were detected. This data provided further evidence that the ‘TrN’ cells might be ILC given other known populations of Id2-expressing cells were gated out by surface markers. We considered this data supportive but not definitive.*

 Question 4: It would be interesting to digest the lung with the ear digest protocol and then analyse the cell surface markers examined in Figure 3 (CD25, ICOS, KLRG-1, MHCII and ST2), to ascertain if the digest protocol used affects the expression of these markers, particularly given the large differences seen in expression of these markers between lung and ear skin. 

*Answer 4: We thank the reviewer for this suggestion and have performed these experiments and included the data within the revised supplementary figures (Supplementary Figure 5 and 6). These new data indicate that expression of both CD25 and KLRG-1 are reduced on cells from the lung when digested with the ear protocol. This then in turn indicates that the ILC2 phenotype reported in the ear is influenced by the digestion protocol and we have tried to make this point more clear in the text. We did try other means of digesting the ear tissue, but the yield was very poor.  We have added this text to the discussion:*

*‘We demonstrate that by increasing the digestion of lung tissue, the frequency of GATA-3 ^+^ ILC2 isolated from the tissue is reduced and the proportion of ILC lacking expression of GATA-3, T-bet and ROR* *g* *t is increased. Thus we conclude that whilst ILC lacking expression of the three group defining transcription factors may exist, care must be taken in interpreting data from digested tissue and this population can certainly be ‘contaminated’ by ex vivo changes to known ILC populations during their isolation and analysis. Whilst ILC progenitor populations within different tissues remain to be fully characterised, these observations highlight how the manner in which different tissues are digested can impact on our ability to properly identify different ILC populations.*

*Whilst we have tried to phenotype ILC populations isolated from different tissues, our data again highlights the potential caveats to studying phenotypic markers following substantial digestion of tissue. Whilst we could detect a robust ILC2 population in ear skin following the digestion protocol used, we are left unable to comment on the expression of CD25 and KLRG-1 at the protein level due to clear loss of these markers during cellular isolation. We do provide evidence that ILCs can still be clearly distinguished since expression of key lineage markers were not lost. ‘*

 Question 5: The expression of markers such as CD25, KLRG1 and ST2 are shown in this report to be expressed at different levels on ILC2s in skin versus ILC2s in lung and gut. The potential reasons behind this differential expression could be addressed more fully in the discussion.

*Answer 5: As outlined above, the experiment suggested by the reviewer provides evidence that the low CD25 and KLRG-1 expression is due to the digestion of the ear and we have made this clearer in the text. We did not find evidence that ST2 expression is lost during the digestion, indicating that ILC2 in the ear skin do indeed lack ST2 expression.*

 Question 6: The data showing increased populations of ILC2 and ILC3 in CD80/86 deficient mouse gut, but lower numbers of ILC3s in mesenteric lymph nodes is intriguing, but it is unclear if this is caused by the lack of CD80/86 signals acting directly on ILCs, or if it is due to a lack of activated T cells in the absence of CD80/86. It would therefore be interesting to analyse ILCs in other transgenic mice where T cell activation is impaired to determine if ILC populations are affected by the presence of activated CD4 or CD8 T cells. Given that the ILC3 population is increased in the lamina propria and decreased in the mesenteric lymph node, it would also be interesting to determine if the increase in these ILC populations in the lamina propria is due to increased turnover, or increased trafficking of these cells or their precursors.

*Answer 6: We agree with the reviewer that further analysis of ILC in different mice lacking costimulatory molecules might be interesting as well as exploring the mechanisms behind such changes. We think this is beyond the scope of this current study and perhaps conditional knockout mice may be more informative should the models become available.*
